# Disruption of Specific RNA-RNA Interactions in a Double-Stranded RNA Virus Inhibits Genome Packaging and Virus Infectivity

**DOI:** 10.1371/journal.ppat.1005321

**Published:** 2015-12-08

**Authors:** Teodoro Fajardo, Po-Yu Sung, Polly Roy

**Affiliations:** Department of Pathogen Molecular Biology, Faculty of Infectious and Tropical Diseases, London School of Hygiene and Tropical Medicine, London, United Kingdom; Purdue University, UNITED STATES

## Abstract

Bluetongue virus (BTV) causes hemorrhagic disease in economically important livestock. The BTV genome is organized into ten discrete double-stranded RNA molecules (S1-S10) which have been suggested to follow a sequential packaging pathway from smallest to largest segment during virus capsid assembly. To substantiate and extend these studies, we have investigated the RNA sorting and packaging mechanisms with a new experimental approach using inhibitory oligonucleotides. Putative packaging signals present in the 3’untranslated regions of BTV segments were targeted by a number of nuclease resistant oligoribonucleotides (ORNs) and their effects on virus replication in cell culture were assessed. ORNs complementary to the 3’ UTR of BTV RNAs significantly inhibited virus replication without affecting protein synthesis. Same ORNs were found to inhibit complex formation when added to a novel RNA-RNA interaction assay which measured the formation of supramolecular complexes between and among different RNA segments. ORNs targeting the 3’UTR of BTV segment 10, the smallest RNA segment, were shown to be the most potent and deletions or substitution mutations of the targeted sequences diminished the RNA complexes and abolished the recovery of viable viruses using reverse genetics. Cell-free capsid assembly/RNA packaging assay also confirmed that the inhibitory ORNs could interfere with RNA packaging and further substitution mutations within the putative RNA packaging sequence have identified the recognition sequence concerned. Exchange of 3’UTR between segments have further demonstrated that RNA recognition was segment specific, most likely acting as part of the secondary structure of the entire genomic segment. Our data confirm that genome packaging in this segmented dsRNA virus occurs via the formation of supramolecular complexes formed by the interaction of specific sequences located in the 3’ UTRs. Additionally, the inhibition of packaging *in-trans* with inhibitory ORNs suggests this that interaction is a bona fide target for the design of compounds with antiviral activity.

## Author Summary

Bluetongue virus (BTV) is an economically important pathogen of ruminants that belongs to a group of viruses whose genome consists of multiple segments of double-stranded RNA. In order for the virus to synthesize viable and infectious progeny, a precise set of the 10 newly replicated BTV segments must be selected for packaging into each new virus particle. How the virus is able to select its own genomic strands from the vast array of cellular RNAs is not clearly understood. One possibility is that that BTV segments harbours an interaction signal that allows them to be sorted and packaged as a set. Correct identification of these signals has basic and applied implications for a possible target of antiviral therapeutics through inhibition of genome sorting and packaging process. Here we showed that a series of short oligonucleotides (ORNs) complementary to multiple sites on the BTV RNA prevented the growth of viable virus in infected cells. ORNs positive for inhibition in virus growth also prevented the genomic RNA to be packaged in an *in vitro* packaging assay. Moreover, when these same targeted sequences were deleted or mutated in viral genome, viable virus recovery was abolished. Exchanging the terminal sequences between segments failed to recover virus confirming that such changes are deleterious to virus viability. These studies have identified specific regions and sequences key to genome packaging in dsRNA viruses and viability. The specific genome packaging sequences targeted by inhibitory activities of ORNs are bona fide drug target which, as a mechanism common amongst all serotypes, may represent an Achilles’ heel for the development of virus therapeutics.

## Introduction

Bluetongue is a vector-borne hemorrhagic disease of livestock and is responsible for considerable economic losses to international livestock industries [1, 2]. The disease is caused by Bluetongue virus (BTV) a non-enveloped virus (a member of *Reoviridae* family) with a double-capsid icosahedral particle and a double-stranded 10-segmented (S1-S10) RNA genome. During virus entry into the cells, the outer capsid of all members of the family including BTV, disassembles from the inner capsid (termed the “core”), which remains intact. The core synthesizes transcripts that are translated into viral proteins, and act as templates for synthesis of genomic dsRNAs [3–5]. However, recent data demonstrated that the ssRNA templates are packaged prior to synthesis of genomic dsRNA [6]. Each BTV RNA segment encodes for one protein except S9 and S10, which encode for two proteins [7, 8]. Based on their size, the 10 segments are classified as large (S1-S3), medium (S4-S6) and small (S7-S10). The 5’ untranslated region (UTR) of each of the ten segments of BTV varies in length from 9 nucleotides for S4 to 35 nucleotides for S6. The 3’ UTRs of each segment also vary in length, being generally longer than the 5’ UTRs and contain a highly conserved hexanucleotide sequence [9]. Due to this, the 3’UTR of each segment have long been thought to contribute to the complex process of RNA sorting and encapsidation and evidence has recently been obtained suggesting that the process of individual recruitment of RNA is likely to be initiated by S10 which then recruits other RNA segments in sequential order, from smaller to larger [6, 10]. It has also been hypothesized that the 3’ and 5’ UTR stem loop and hairpin loop structures interact and mediate a conformational change that also relate to packaging [11]. However, direct evidence for RNA-RNA interactions and the involvement of the 3’UTR in sorting and packaging of the BTV genomes have not been demonstrated to date.

To investigate the mechanism of BTV genome packaging, a series of short single-stranded synthetic oligoribonucleotides (ORNs) complementary to specific RNA motifs of different genomic segments was used as competitive agents based on predicted RNA secondary structure. Designed ORNs were found to be inhibitory for virus replication in cell culture but did not inhibit *in vitro* protein synthesis. The inhibitory effects were further investigated using novel *in vitro* assay systems able to detect supramolecular complex formation via specific RNA-RNA interactions. The data is consistent with inhibitory ORNs targeting regions in the 3’ UTR and leading to inhibition of virus replication by competition with RNA complex formation and packaging. The study revealed RNA-RNA interactions driven by the smallest segment, S10 but also by S7 suggesting that specific multi-site interactions between different segments are required to trigger the packaging of BTV RNA segments. Interchanging 3’ UTRs among segments prevented virus recovery, indicating that the newly mapped packaging/ RNA interaction signals on each BTV segments are specific to their resident segment.

## Results

### Oligonucleotides targeting BTV RNA segments affect virus replication

**Fig 1:**
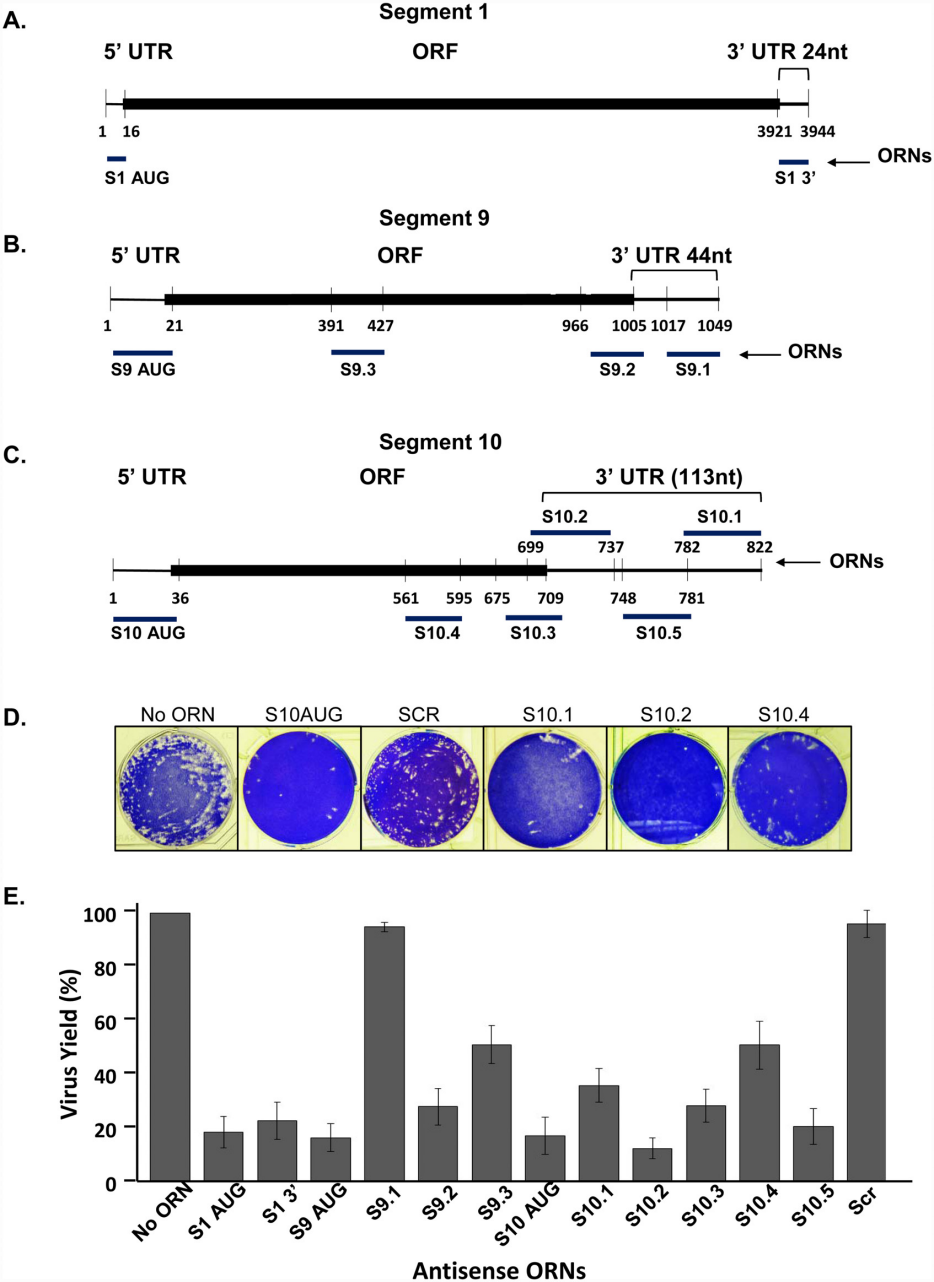
*In vivo* effect of antisense ORNs complementary to S1, S9 and S10 on virus replication. Schematic representation of S1 **(A)**, S9 **(B)** and S10 **(C)** indicating the 5’ and 3’UTRs and the protein coding region (ORF) with the initiation codon (AUG). Positions targeted by the antisense ORNs and 3’UTR length are indicated in each case. **(D)**. Representative examples of plaque assay stained with crystal violet **(E).** Histogram of virus yield in the presence of different ORNs. S1, S9 and S10 or Scr ORNs were transfected to BSR monolayer cells for 3 hours followed by infection with BTV-1 at 0.1 MOI. At 24hpi virus yield was determined by plaque forming units (PFU) as described in Material and Methods. Values (%) represent the mean and standard deviation of the mean (n = 3–5) generated relative to the control (without ORNs) set at 100%.

Our previous data suggested that the 3’ UTRs are essential for packaging of positive sense ssRNAs during BTV assembly and that the packaging is initiated by the smallest segment S10 [10]. We sought to investigate whether small specific antisense oligoribonucleotides (ORNs) targeting the 3’ terminal sequences of these smaller segments would interfere with BTV growth. A set of ORNs complementary to the UTRs of positive sense ssRNA of S9 and S10 were designed based on the predicted RNA secondary structure as no RNA probing data is available for BTV, to date (Fig 1,S1and S2 Figs). For stability and to avoid the cellular immune response, the 2’OH of the ribose of each ORN was modified to 2’O-methyl. The sequences of each ORN are presented in Table 1.

**Table 1:** The 2’O-methyl modified antisense oligoribonucleotides (ORNs) used for *in vivo* and *in vitro* studies.

ORN	Antisense sequence 5’– 3’ (2’O-methyl modified)	Binding region	Lengthnt
**Segment 1 (3944 nt)**			
**S1 AUG**	ACCAUUGCAUUUUAAC	S1 5’ UTR + start codon nt16-1	**16**
**S1 3’**	GUAAGUGUAAUGCGGCGCGUGCUC	S1 3’ UTR nt3944-3921	**24**
**Segment 9 (1049 nt)**			
**S9AUG**	UGACAUAUGCGAUUUUUUAAC	S9 5’ UTR + start codon nt21-1	**21**
**S9.1**	GUAAGUGUAAAAUCGCCCUACGUCAAGAAGGUA	S9 terminal 3’ UTR nt1049-1017	**33**
**S9.2**	UUAGAGGUGAUCGAUCAAAUGCAGGAACUCCGUUUUCACA	S9 coding region (3’ term + stop codon) nt1005-966	**40**
**S9.3**	CUUCUGUUAGAACUACCCAUCUUCCUCCAUUCGCUCC	S9 coding region (5’ term) nt427-391	**37**
**Segment 10 (822nt)**			
**S10AUG**	AUCAGCCCGGAUAGCAUGGCAGCGACACUUUUUAAC	S10 5’ UTR + start codon nt36-1	**36**
**S10.1**	GUAAGUGUGUAGCGCCGCAUACCCTCCCCCGUUAGACAGCA	S10 terminal 3’ UTR nt 822–782	**41**
**S10.2**	CCUCGGGGCGCCACUCUACCUACUGAUCUUAGGUUAAUG	S10 stop codon to 3’ UTR nt 737–699	**39**
**S10.3**	UUAGGUUAAUGGUAAUUCGAAACCAUCUAGCGGGA	S10 coding region (3’term + stop codon) nt709-675	**35**
**S10.4**	AAUUUGCUGGUUCAAGCUUCUCUCGCUUUUUGCGC	S10 coding region (3’ term) nt595-561	**35**
**S10.5**	GTAGGAGTCTGCATCGTGAGATCAACCACTCTAC	S10 3’UTR nt748-781	**34**
**Scrambled (SCR)**	UGCUAUUACCAUGCUACAGAUGUAAGUGAU	scrambled sequence	**30**

The ORN name, sequence (5’-3’), length, target BTV RNA segments regions are listed.

Six ORNs complementary to different regions including the 3’ conserved terminus of the S10 (Fig 1C) were designed to interfere with the RNA structures (shown in S1–S3 Figs), and three of which encompassed the entire length of the S10 3’ UTR. S10.1 was complementary to the 3’ terminal 41 nt (nt822-782) including the conserved sequence, 39 nt of S10.2 was complementary to nt737-699, including the stop codon, and the 34 nt of S10.5 complimentary to nt781-748, the region between S10.1 and S10.2. The other ORNs targeted the structure outside of the 3’UTR; S10.3 to the terminal 35 nucleotides of the coding region (ORF), S10.4 in the ORF (nt595-561) and S10AUG, the initiation codon. For segment 9 (S9), the 3’ UTR consists of 44 nts (nt1049-1006), and thus, three ORNs encompassed part of the UTR and part of the 3’ ORF (Fig 1B). One ORN (S9.1) was complementary to the 3’ terminal 33 nt (nt1049-1017), while ORNs S9.2 and S9.3 were complementary to the last 40 nucleotides of the coding region including the stop codon (nt1005-966) or the middle section of the coding region (nt427-391), respectively. In addition, for positive controls, ORNs complementary to the 5’ UTR regions including the AUG codons of both S9 (S9 AUG) and S10 (S10 AUG) (Fig 1B & 1C; Table 1) and a SCR sequence of 30 nucleotides were also synthesized. The secondary structures of S9 and S10 and position of ORNs are shown in S1–S3 Figs.

For *in vivo* assay, the concentration of ORNs was first optimized and subsequently BSR cells were transfected with 1.5 μM of each ORNs and Scr ORNs. At 3 hours post-transfection (hpt), cells were infected with BTV-1 of MOI of 0.1 and virus titres were monitored 16 hpi. Analysis of each ORN-transfected BSR cells followed by infection with BTV-1 showed S10 ORNs had a negative effect on virus yield albeit to a varying degree. Specifically, ORN S10.2 was the most inhibitory where virus yield was reduced by ~90% while S10.3 had also a significant effect on virus replication with ~70% reduction in comparison to that of the control (Fig 1D). These ORNs were complementary to the 3’ end of the coding region (S10.3) and beginning of the 3’ UTR (S10.2). Secondary structure prediction of S10 revealed the S10.2 ORN was complementary to a GC rich hairpin loop, a bulge and a double-stranded region (S1 Fig). S10.1 ORN, which covered the terminal 41 nts of 3’UTR, also had a significant inhibitory effect on virus yield (~70% reduction), consistent with our previous report [11]. In contrast, ORN S10.4, which targeted part of the coding region (nt595-561) was less inhibitory. That all S10 antisense ORNs had some interference activity on virus replication is consistent with the smallest BTV RNA segment playing a crucial role in virus replication, as reported [10]. In contrary to S10, S9.1 ORN, complementary to the last 33nt of S9 3’ UTR, had only a marginal effect on virus recovery (Fig 1D). However, virus growth was reduced by ~80% in the presence of S9.2, which encompasses the 40 terminal nucleotides (UTR+ORF) and to a lesser extent, ~50%, by S9.3 ORN (ORF only). As expected, the presence of the control ORNs, S10 AUG or S9 AUG, virus growth was severely reduced. On the contrary, parallel assays with scrambled sequences showed no inhibitory effect on virus replication. Further, no cell toxicity was observed up to 48 hrs of incubation of BSR cells with different concentrations of Scr ORNs (0.1–2.5μM) followed by staining the viable cells (S4 Fig), indicating that the effects of ORNs observed on BTV infected cells were specific to BTV replication.

Based on the inhibitory results of the ORN targeting the 3’UTR, we also investigated the effect of an ORN that encompasses an entire 3’UTR. We selected S1 as it possesses the shortest 3’UTR (24 nt) of all BTV RNA segments. To this end, we designed an ORN complementary to the entire length of the 3’UTR and, as positive control, another to the 5’UTR including the AUG codon (Fig 1A). Virus titer was reduced to ~20% in the presence of the S1 3’ ORN as compared to control without ORN and was similar to that of the 3’ UTR ORNs of S10 (Fig 1D).

**Fig 2:**
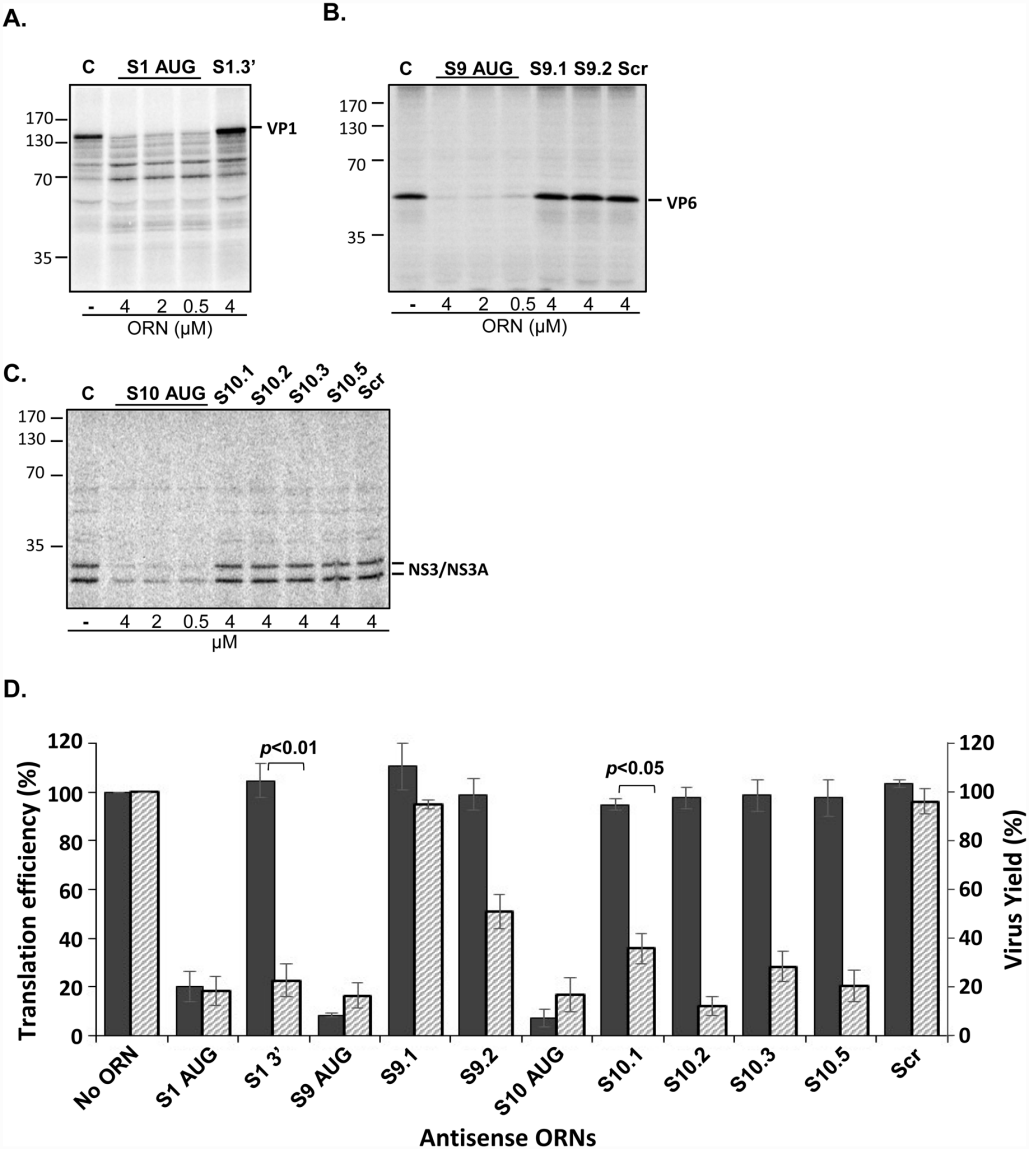
Translation efficiency of BTV mRNA in the presence of ORNs. Synthesized VP1 (S1) **(A)**, VP6 (S9) **(B)** and NS3/NS3A (S10) **(C)** viral proteins in the absence or presence of antisense ORNs in varying concentration (μM). Positions of molecular mass standards are indicated in kDa. **(D)** Histogram of virus yield and cell-free translation in the presence of different ORNs are indicated. Translation efficiency values were calculated by densitometry as the ratio of the translated product relative to the ‘No ORN’ control, set as 100%. Virus yield was expressed as the reduction of the number of plaques (PFU). Values (%) represent the mean and standard deviation of the mean (n = 3).

Antisense oligonucleotides could trigger steric blocking of viral mRNA and thereby perturb the translation of viral mRNAs, therefore we examined if the inhibition of virus growth was due to the interfering effect of ORNs on the efficiency of virus protein expression. To validate this, we performed a cell-free translation in the presence or absence of ORNs complementary to the initiation codons of S1 (VP1), S9 (VP6) and S10 (NS3/NS3A) or the 3’ UTR region. Analysis of translated products showed that VP1, VP6, NS3/NS3a viral proteins were efficiently translated in the presence of ORNs complementary to the 3’UTR regions (Fig 2A–2D). In contrast, a marked reduction of encoded protein levels were observed in the presence of S1, S9 and S10 AUG ORNs, respectively (Fig 2A–2D), consistent with the *in vivo* data (Fig 2D). Conversely, scrambled ORN control did not inhibit the translation of S9 and S10 mRNAs (Fig 2B–2D), indicating sequence specificity of the ORNs to block their target regions. The significant inhibition of virus replication in the presence of 3’UTR ORNs *in vivo* in contrast to the efficient BTV protein synthesis *in vitro* suggests a mechanism of action whereby 3’UTRs of BTV RNA segments are important in virus replication.

### Complex networks of ssRNA segments and disruption by ORNs

**Fig 3:**
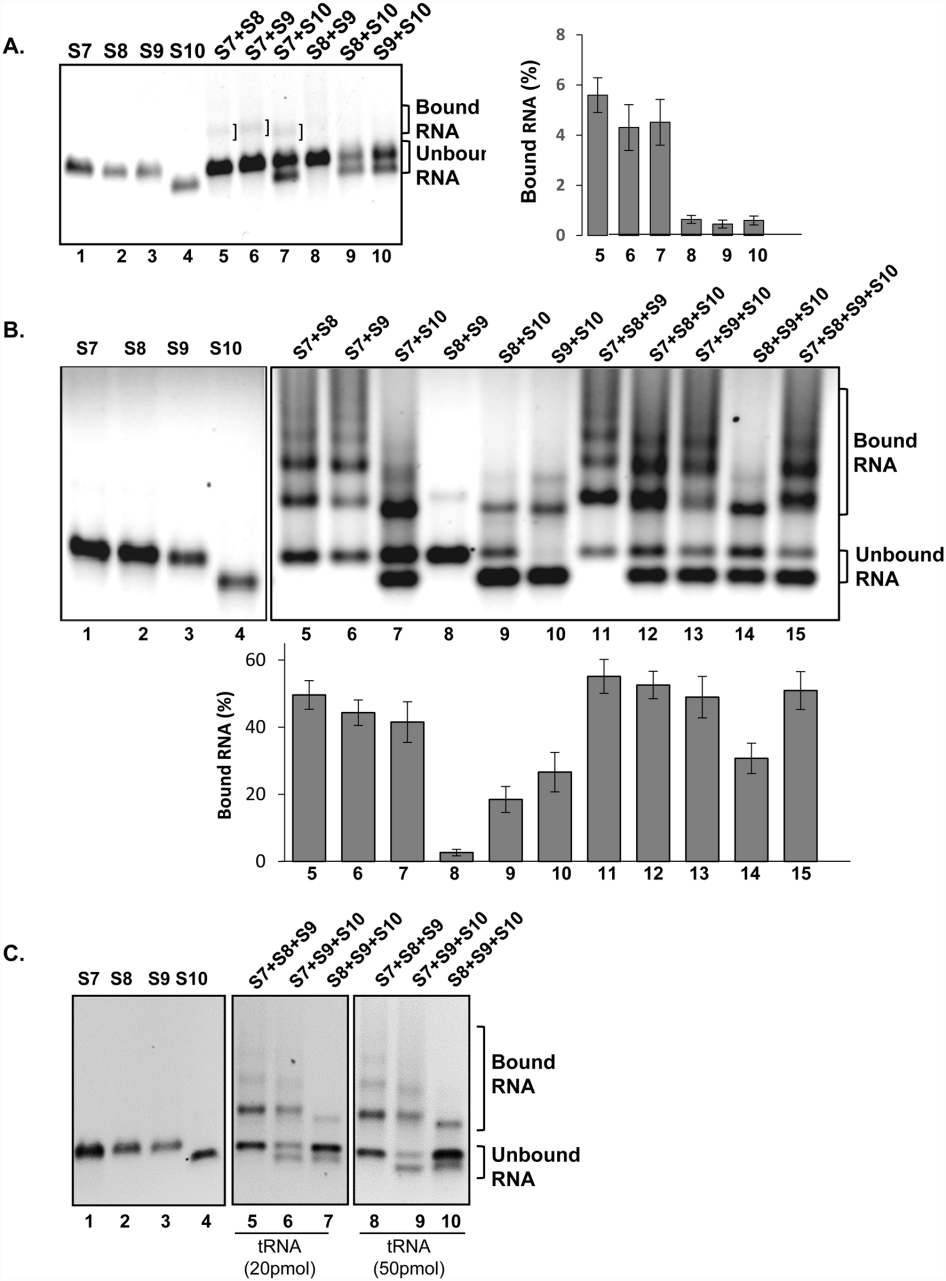
RNA-RNA interactions between small BTV segments. **(A)** Independently transcribed and purified RNA segments (lanes 1 to 4) were heated for two minutes prior incubation in pairs (lanes 5 to 10) as indicated. The mobility shift was analysed by native agarose gel. Interactions are indicated on the right. **(B)** Simultaneous co-transcription of multiple segments (lanes 5 to 15) as indicated. The positions of the retarded complex and free RNA are indicated on the right (upper panel). Purified and co-transcription reactions, RNA-RNA complexes were quantified by densitometry and expressed as percentage of the ratio of bound and unbound RNA (lower panels of **A** and **B**). Values (%) are from the mean and the standard deviation of >3 independent assays (n = 3–5). Individually transcribed segments are shown (lanes 1 to 4, upper panels **A** and **B**) as controls. **(C)** For specificity, multiple co-transcription in the presence of different amounts of yeast tRNA are shown. The position of the bound (retarded RNA complexes) and unbound free RNAs are indicated.

Since ORNs inhibited virus replication but did not affect protein translation, ORNs have most likely interrupted the RNA-RNA interactions and packaging during virus replication. To investigate these it was necessary to visualize the formation of RNA complexes in absence of ORNs. We modified an electrophoretic mobility shift assay (EMSA) for visualization of RNA complexes from RNA segments of dsRNA virus, which allowed us to visualize RNA interactions and large complex formation following two different experimental approaches: (1) Co-incubation of two purified ssRNA segments for hybridization assay and (2) Co-transcription of T7 cDNA copies of segments in pairs or in combinations of 3 or 4. The EMSA analysis of co-incubation products exhibited shifted weak bands for combinations of S7+S8, S7+S9 and S7+S10 (Fig 3A, lanes 5 to 7) indicating that S7 interacts with each of the other three small segments to form a complex. Other RNA segment combinations did not show any distinct retarded bands (Fig 3A, lanes 8, 9, 10) even in the presence of S10. In contrast to co-incubation, distinct retarded bands appeared when two segments were co-transcribed from T7 cDNAs (Fig 3B, lanes 5 to 10), except S8+S9 (Fig 3B, lane 8), suggesting that RNA segments were interacting during or soon after they were synthesized and that the presence of either S7 or S10 stimulated the complex formation.

In three or four co-transcribed RNA segments, stronger intermolecular interactions were detected with additional shifted bands in each case and the amount of free, unbound RNA was also less than when only two segments were co-transcribed (Fig 3). Further, the appearance of additional RNA complex were noticeable when S7 and S10 were present in the reaction (Fig 3B, compare lanes 5 to 10 and 11 to 14) suggesting that although S10 plays a key role in bringing the smaller segments together, S7 is also necessary to form a RNA network of all four segments. The addition of S10 in a reaction of S7, S8 and S9 also led to stronger retarded bands (Fig 3B, compare lanes 11 and 15) which strengthens the role of S10 in the intermolecular interaction. It was evident that the presence of S7, which has the second longest 3’ UTR after S10, (Fig 3B, compare lanes 8 to 10 and 11 to 13, also compare lanes 14 to 15) is crucial for strong complex formation. Table 2 summarizes the results obtained from the RNA-RNA interaction studies of purified and co-transcribed segments.

**Table 2:** Summary of RNA-RNA interactions studies.

Summary of RNA-RNA interactions between segments (% of bound RNA)				
Two RNA segments	Interactions of purified RNA	Interactions of co- transcribed RNA	Three & Four RNA segments	Interactions of co- transcribed RNA
S7+S8	5.6 +/- 0.8	50 +/- 4	**S7+S8+S9**	55 +/- 5
S7+S9	4.3 +/- 0.8	44 +/- 4	**S7+S8+S10**	53 +/- 4
S7+S10	4.5 +/- 0.8	42 +/- 6	**S7+S9+S10**	49 +/- 6
S9+S10	0.6 +/- 0.2	27 +/- 6	**S8+S9+S10**	31 +/- 4
S8+S10	0.4 +/- 0.1	18 +/- 4	**S7+S8+S9+S10**	51 +/- 5
S8+S9	0.4 +/- 0.1	3.0 +/- 0.9		

Left panel: Interactions between two RNA segments of purified or co-transcribed RNAs. Right panel: Interactions among three or four segments by co-transcription. Values (%) are from mean and standard deviation of >3 independent experiments (n = 3–5).

The specificity of RNA-RNA interactions was tested in the presence of non-specific competitor yeast tRNA at 20 to 50 fold molar mass excess and the level of complex formation was not significantly reduced (Fig 3C) indicating that interactions between RNA segments were sequence specific.

**Fig 4:**
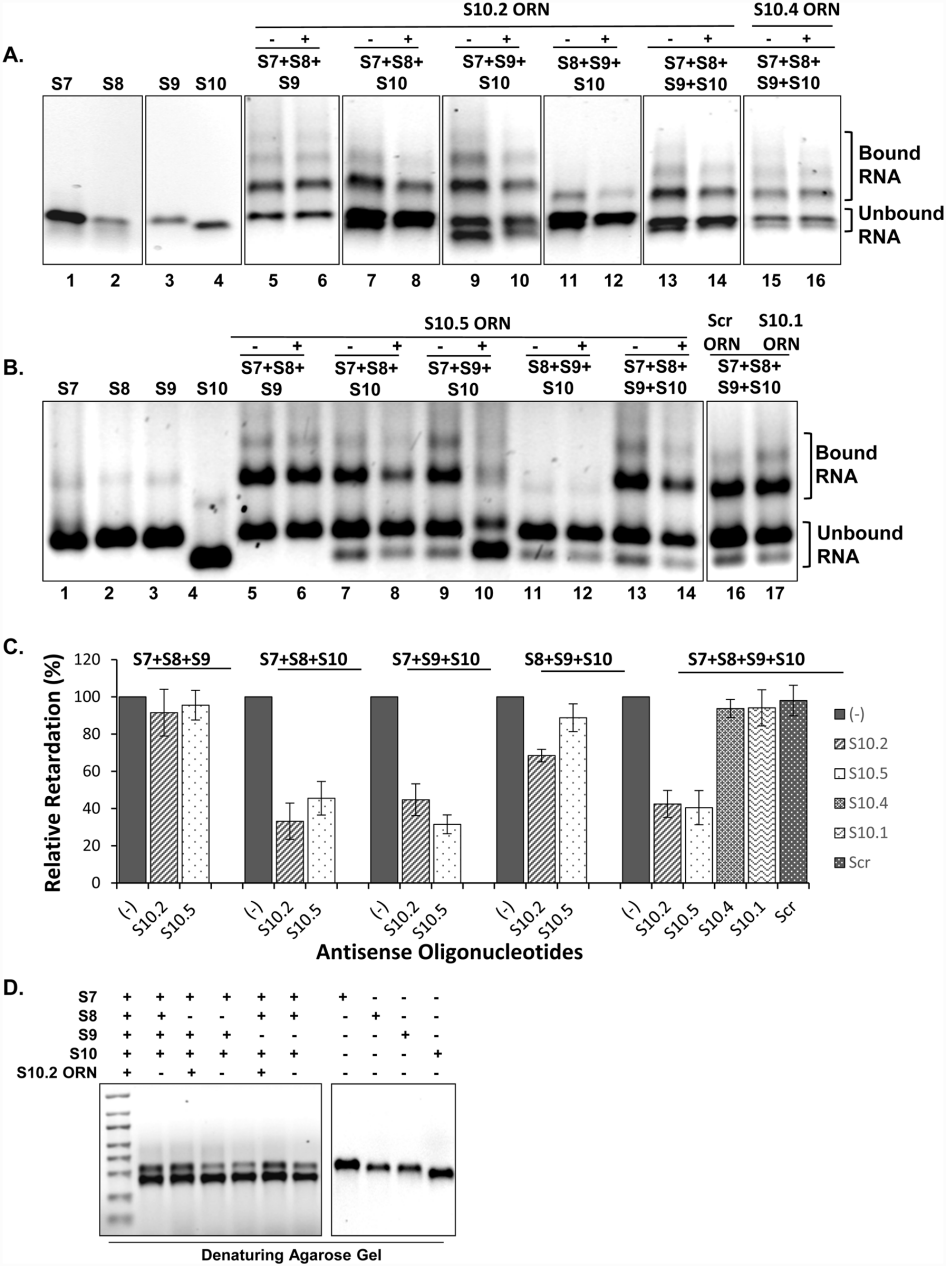
Mobility shifts of RNA complexes in the presence of ORNs. **(A)** Combinations of three or four BTV RNA segments were co-transcribed in the presence (+) or absence (-) of S10.2 (lanes 5 to 14) or S10.4 (lanes 15 and 16) ORNs. The bound RNA complexes and unbound RNAs are indicated (upper panel) and their quantifications are shown in histogram (lower panel). **(B)** The effect of Scr, S10.1 and S10.5 ORNs on RNA complex formation was similarly analysed and presented. (**C**). Histogram of the percentage of the RNA complex in each lane of A and B was determined in relation to the total mass of input RNA. The RNA complexes in presence of ORNs were normalized relative to the control complexes without ORNs. Values (%) represent the mean and standard deviation of >3 independent assays (n = 3–5). **(D)** RNAs from co-transcription reactions in the presence or absence of S10.2 analyzed on a 1% denaturing agarose gel.

To determine if the RNA complexes following co-transcription of multiple segments could be disrupted by ORNs targeting the S10 3’UTR, all four small RNA segments or different combinations of three (S7+S8+S9, S7+S8+S10, S7+S9+S10, S8+S9+S10) were co-transcribed in the presence or absence of 20 pmol of either S10.2 and S10.5 ORNs (most inhibitory ORNs in virus replication) or S10.4 ORN (non-inhibitory ORNs targeting the ORF) (see Fig 1A, 1B & 1C). EMSA data showed that RNA complexes in the presence of S10.2 and S10.5 were reduced up to four fold when compared to the control RNA complexes (Fig 4A & 4C) but not with S10.4. When the same reaction was performed in the absence of target RNA S10 (i.e.S7+S8+S9 only) the RNA complexes were not affected by the presence of S10.2 or S10.5 ORNs (Fig 4A & 4B, lanes 5–6).The RNA complex formed by S8, S9 and S10 (but not S7) in the presence or absence of S10.5 ORN was too weak to ascertain the inhibition activity (Fig 4B, lanes 11–12). These data suggest that the intermolecular interactions among the four smaller segments requires both S10 and S7 and interactions initiated by the S10 and S7 could be specifically disrupted by S10.2 (39 nt) or S10.5 (34 nt). These results emphasize that sequences encompassing by these two ORNs at the 3’UTR downstream of the S10 stop codon are involved in intermolecular RNA-RNA interaction. The S10.2 ORN was designed to target the GC rich hairpin loop, bulges and duplex while S10.5 targeted a duplex and hairpin loop (S1 Fig). Results also suggested that the terminal 41 nt of S10 3’ UTR (S10.1) or the last 35 nt in the S10 coding region (S10.4) are not essential for interactions.

The specificity of the ORN to inhibit RNA-RNA interactions was further demonstrated by Scr ORN, which had no effect on RNA complexes (Fig 4B, lane 16). The integrity of the transcribed RNAs was confirmed by denaturing gel analysis of the co-transcribed ssRNA segments which showed the position of the transcribed RNAs of each segment (Fig 4D). The presence of distinct bands of complexes and unbound RNAs as detected by EMSA demonstrating the RNAs were transcribed by these plasmids in presence of ORNs. Hybridization assay also showed that ORN S9 AUG and ORN S9.2 hybridized with S9 mRNA, while ORN S10 AUG and ORNs S10.2, S10.3, S10.5 annealed to S10 mRNA. No hybridization with Scr control was detected when incubated with S10 and S9 mRNAs (S10 Fig).

### Identification of regions in S10 responsible for interactions with other segments

**Fig 5:**
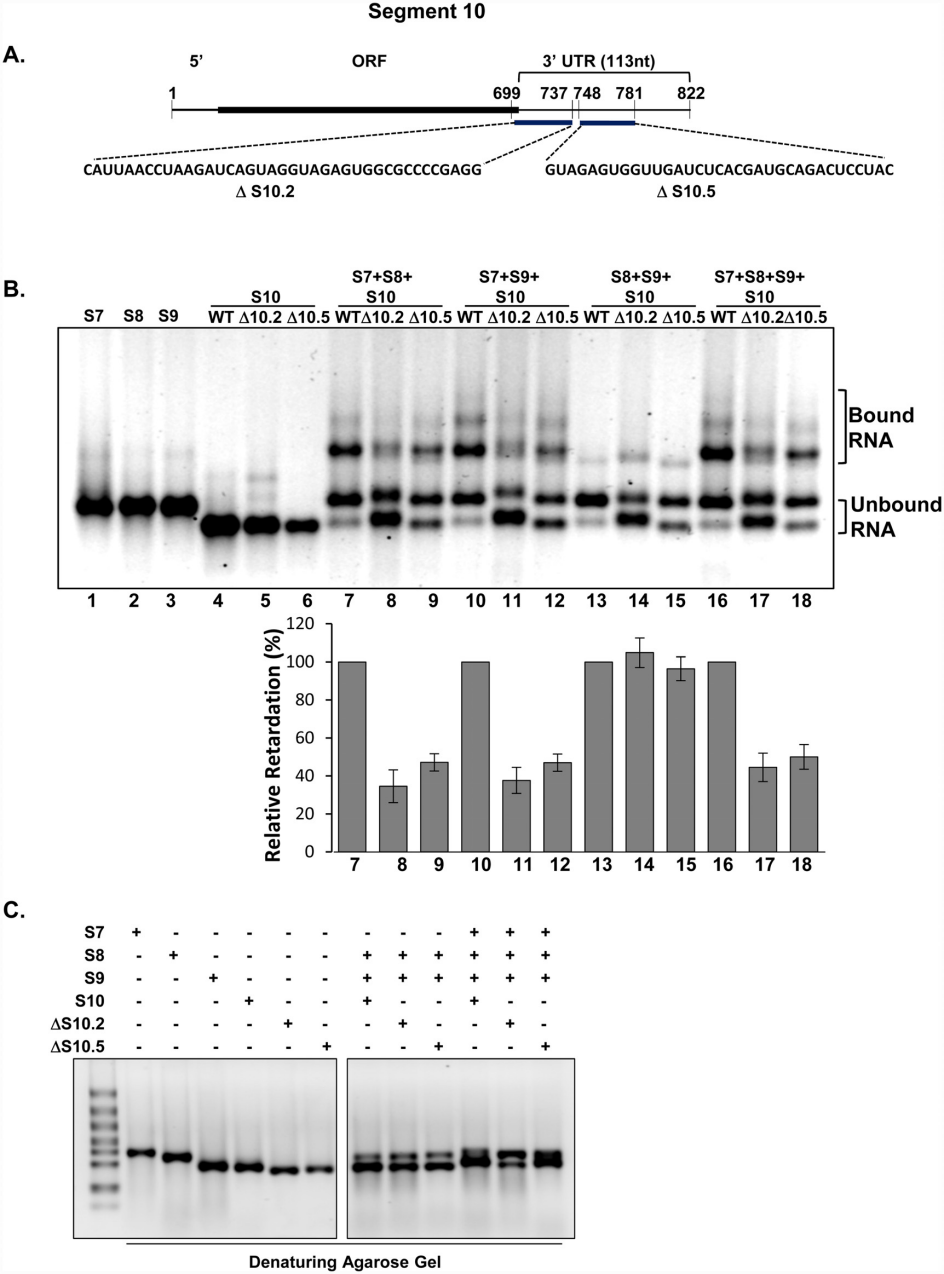
Effect of deletions in S10 on RNA-RNA interactions. **(A)** Schematic representation of S10 depicting deleted sequences (ΔS10.2 and ΔS10.5) as indicated. **(B)** Mobility shift assay of co-transcription complexes in the presence of ΔS10.2 and ΔS10.5 mutants (lanes 7 to 18). Positions of retarded complexes and free RNAs are indicated and quantifications of bound to unbound RNAs are shown (lanes 7–18, lower panel). The RNA complexes in each lane with S10 WT or each mutant were determined against the total mass of input RNAs as (%). The RNA complexes with S10 mutants were normalized relative to the complexes formed with the WT S10. Values (%) represent the mean and the standard deviation of >3 independent assays (n = 3–5). **(C)** Simultaneous or individual RNA transcriptions in the presence or absence of ΔS10.2 or ΔS10.5 analyzed in a 1% denaturing agarose gel.

The decreased RNA complex formation in the presence of S10 3’UTR ORNs prompted us to explore the key regions in S10 RNA responsible for recruiting other segments and complex formation. Deletion mutants in S10 which spanned the sequence of inhibitory ORN were constructed and used in the RNA-RNA interactions with other segments (Fig 5A). The regions of deletion mutations are shown in S5 Fig. Up to four fold reductions in RNA complex formation were observed with each of S10.2 and S10.5 deletion mutants in combination with S7+S8, S7+S9 and S7+S8+S9 when compared with the reactions with wild-type S10 (Fig 5B). As previously, in the absence of S7, no complex was detectable when S8 and S9, were used with either S10 or S10 mutants. The RNA structures of deletion mutants showed that when target regions of S10.2 and S10.5 were deleted, the hairpin loops and bulges were either significantly altered or absent compared with the wild-type structure (S5 Fig). This was consistent with the results obtained when using ORNs to inhibit RNA interactions (see Fig 4A & 4B) suggesting multiple sites in S10 are necessary for sorting and recruitment of other segments. The reduction of RNA complex formation in a reaction with deletion mutants S10.2 and S10.5 suggests the key role of S10 in recruiting other segments for complex formation and the importance of the sequence in the S10 3’UTR for intermolecular interactions which become more evident in the presence of S7 in the interaction reaction. The integrity of transcribed RNAs was confirmed by denaturing gel electrophoresis analysis of the co-transcribed wild-type and mutant RNA segments (Fig 5C). The results obtained from RNA-RNA interaction studies in the presence or absence of ORNs and S10 deletion mutants are summarized in table 3.

**Table 3:** Interactions of multiple BTV segments in the presence or absence of ORNs (left panel) and S7, S8, S9 with S10 WT or S10 with deletion mutants (right panel).

RNA-RNA interactions of segments + ORNs (% of relative RNA retardation)				RNA-RNA interactions of segments with S10 WT or S10 deletion mutants (% of relative RNA retardation)			
BTV segments	No ORN	+ S10.2 ORN	+ S10.5 ORN	BTV segments	WT S10	ΔS10.2	ΔS10.5
S7+S8+S9	100	92 +/- 12	96 +/- 8	**S7+S8+S9**	N/A	N/A	N/A
S7+S8+S10	100	33 +/- 10	46 +/- 9	**S7+S8+S10**	100	34 +/- 8	47 +/- 5
S7+S9+S10	100	45 +/- 8	31 +/- 5	**S7+S9+S10**	100	37 +/- 9	47 +/- 5
S8+S9+S10	100	68 +/- 3	89 +/- 15	**S8+S9+S10**	100	105 +/- 9	96 +/- 6
S7+S8+S9+S10	100	42 +/- 7	40 +/- 9	**S7+S8+S9+S10**	100	44 +/- 7	50 +/- 6

Values (%) are the mean and standard deviation of >3 independent experiments relative to control (no ORN or S10 WT) set at 100% (n = 3–5).

### Specific ORNs inhibit BTV RNA packaging during capsid assembly

**Fig 6:**
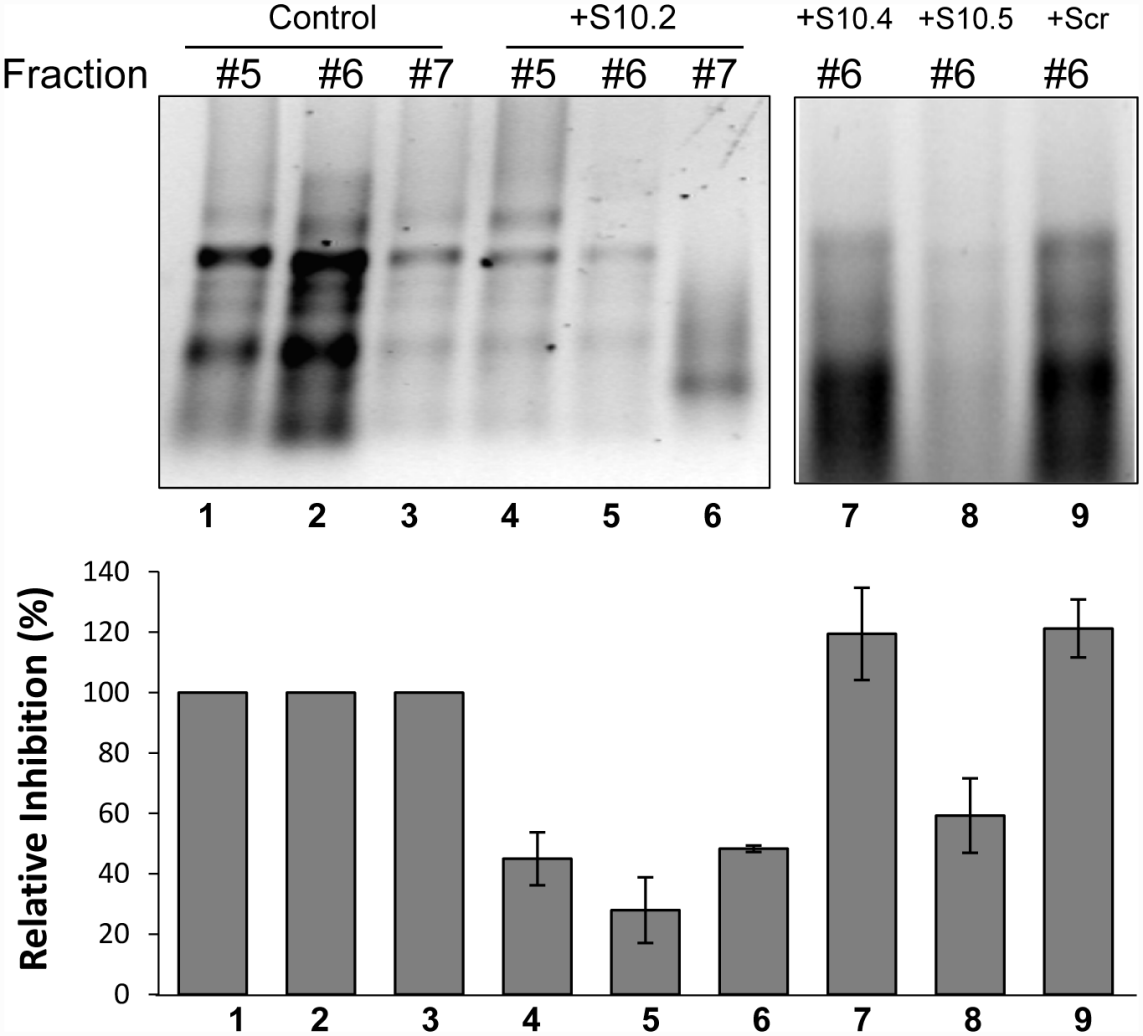
Effect of ORN on RNA packaging in *cell-free* assembly assay. ^35^S-labelled *in vitro* assembled BTV complexes were fractionated in a continuous sucrose gradient [6]. (Upper panel): Fractions #5, #6 and #7 from *cell-free* assembly (CFA) reactions in the absence (+control, lanes 1 to 3) or presence of 20 pmol S10.2 ORN (lanes 4 to 6) alongside with fraction #6 in the presence of S10.4 ORN (lane 7) S10.5 (lane 8) and Scr ORN (lane 9) were analysed on 1% denaturing agarose gel. Packaged RNAs were determined by densitometry. Lower panel represents the mean values (%) of total packaged ssRNAs in the presence of ORNs calculated relative to the control reaction (without ORNs) set at 100% (n = 3). The synthesized ^35^S-labelled BTV subcore and transcription complex protein profile are shown in S8 Fig.

To understand further the mechanism of action of S10.2 and S10.5 ORNs and to determine if the inhibitory effects of ORNs on virus growth and RNA-RNA interactions were directly related to BTV RNA packaging during capsid assembly, we utilized a unique cell-free core assembly system that has been successfully used to understand the order of BTV capsid assembly and the genomic segment packaging previously [6, 10]. For this study, S10.1, S10.2, S10.5, S10.4, S10 AUG and Scr ORNs were annealed to S10 transcripts prior to mixing with the remaining 9 BTV ssRNA segments and subsequently incubated with pre-translated transcription complex (VP1, VP4 and VP6) before adding two major core proteins, VP3 and VP7 sequentially. After removing the unpackaged ssRNAs by RNase treatment, the putative *in vitro* assembled cores were purified by centrifugation on a sucrose gradient followed by fractionation, ssRNAs isolation and analysis as described in Methods and Materials. Only S10.2 or S10.5 ORNs, (in fraction 6) inhibited the packaging of 10 BTV ssRNA with ~80% and ~60% reduction respectively (Fig 6, lanes 4–6 & 8). The inhibition of packaged RNAs was not detected in presence of S10.4 and Scr ORNs (Fig 6, lanes 7&9) or with S10.1 and S10 AUG ORNs (S7 Fig). This indicates that by base pairing to the complementary sequences in the S10, both ORNs were capable of inhibition of recruitment and packaging of the not only S10 but all the other 9 segments, possibly due to disruption of RNA-RNA interactions.

To confirm that core proteins were still synthesized efficiently in the cell-free assembly assay, each protein was ^35^S-labeled and the fractionated complex was analyzed by SDS-PAGE. The ^35^S-labelled reconstituted protein products showed the complete set of core proteins, the three proteins of transcription complex (VP1, VP4 and VP6) and the two major core proteins (VP3 and VP7) from fraction no.6 in the presence or absence of S10.2 ORN (S8 Fig) which demonstrated that the transcription complex (TC) and the subcore proteins were efficiently synthesized and assembled and were not hindered in the presence of S10.2 ORN. The effects of different ORNs in RNA packaging by *in vitro* assembly, *in vivo* virus replication, *in vitro* protein synthesis and RNA-RNA interactions are summarized in Table 4.

**Table 4:** Summary of the inhibitory effects of ORNs.

Inhibitory effects of ORNs				
ORN	*In vivo* virus replication	*In vitro* translation	RNA-RNA interactions (4 RNA segments)	*In vitro* RNA packaging
S10.1	**-**	**-**	**-**	**-**
S10.2	**+**	**-**	**+**	**+**
S10.4	**-**	**-**	**-**	**-**
S10.5	**+**	**-**	**+**	**+**
S10 AUG	**+**	**+**	**-**	**-**
Scr	**-**	**-**	**-**	**-**

Plus (+) sign indicates inhibitory effects and negative (-) non-inhibitory effects deduced from different assays performed.

### Virus recovery is inhibited by S10 substitution mutations and chimeric 3’UTR

**Fig 7:**
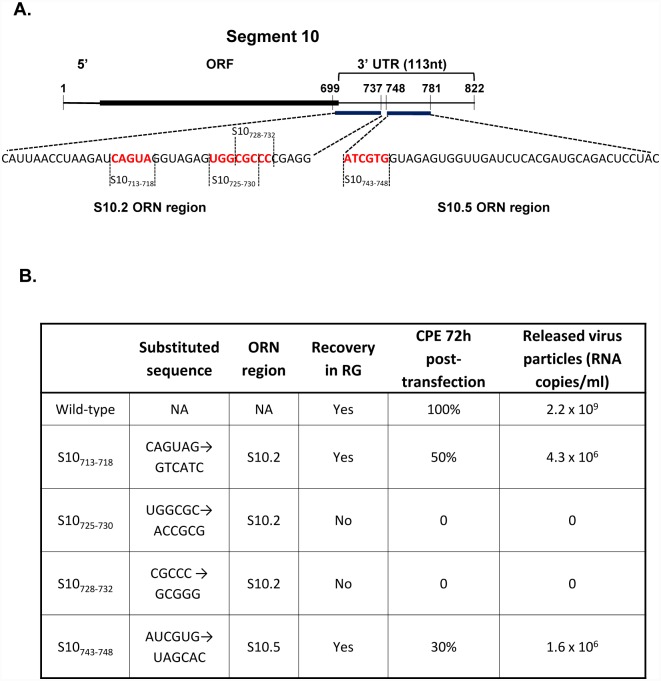
Designed mutations used in reverse genetics system. A. Schematic map of S10 showing the position of substitution mutations on regions encompassed by S10.2 and S10.5 ORNs in the 3’UTR. B. Sequences (5’-3’) of S10 substitution mutants, position of each in relation to different ORNs and their effects on virus recovery by RG system. Percentage of cells exhibiting CPE and RNA copy number of virus particles are indicated. Copy number of genomic segment S6 was used to quantify the amount of released virus particles.

To confirm if the sequences within the identified 3’UTR regions in S10 RNA are important for RNA packaging *in vivo*, four substitution mutations were introduced by targeting five or six nucleotides in the putative binding sites of S10.2 and S10.5 regions at the S10 3’UTR (S6 Fig & Fig 7A). Each mutant S10 ssRNA was used to recover mutant viruses using RG system as described in Materials & Methods [12]. Among the mutants tested, only S10_**713-718**_ (sequence encompassed by ORN S10.2) and S10_**743-748**_ (sequence encompassed by ORN S10.5) (see S5 & S6 Figs) were successfully recovered but exhibited significantly less cytopathic effects (CPE). Further, ~1000 fold less virus particles were detected by qRT PCR in comparison to that of the wild-type at 72 hours post-transfection (Fig 7B & S11 Fig). The nucleotide substitutions in these two mutants were located in the double stranded region of the stem loop structure (S6 Fig). Mutants S10_**725-730**_ and S10_**728-732**_, which encompasses the hairpin loop of the S10.2 region, could not be rescued, consistent with a lethal phenotype.

To investigate further if the identified packaging signals in S10 3’UTR are interchangeable with other segments, 3’ UTR of S8 (see S9 Fig) and S10 (see S1 Fig) were exchanged (S8-UTR10 and S10-UTR8) and chimeric ssRNAs were synthesized. When BSR cells were transfected with each of the chimeric RNA segments together with 9 WT ssRNA segments or all 10 WT ssRNAs as control, only control WT virus was recovered while both chimeric segments failed to recover virus. These data suggest that the packaging signals in the UTRs were not functional when interchanged between different segments.

## Discussion

The exact mechanism by which BTV selects its ten genomic RNA segments among the multitude of other RNAs in the host cytoplasm and packages one copy of each into an assembling capsid to generate an infectious virus particle is not well understood. Recently we have suggested that the 10 RNA segments are packaged through a sequential process by RNA interactions involving the 3’UTRs [10]. In influenza A virus, with a genome of eight discrete negative strand segments, specific interactions have been suggested among the ribonucleoprotein complexes or the eight genomic RNA segments are selected and packaged as an organized supramolecular complex [13, 14]. In *Reoviridae*, with multiple dsRNA segmented genomes and a complex capsid assembly process, the process is challenging although there have been suggestions that genomic RNAs utilize RNA-RNA interactions in the 3’UTRs for assortment and packaging despite no direct evidence being reported to date [15–17]. Current study was therefore aimed to investigate the specific RNA-RNA interactions among the BTV transcripts, which lead to the formation of supramolecular RNA networks and RNA packaging using a range of *in vivo* and *in vitro* experiments sequentially.

Our initial approach in this study, was to utilize short complementary ORNs to assess their effects on virus replication. Several of these ORNs, notably ORNs targeting the 3’ UTRs of S1 and S10, had inhibitory effects on virus growth but not on protein synthesis, suggesting that the inhibition is not at the level of translation and prior to genome encapsidation, but possibly at the stage of genome segment sorting and packaging, consistent with our previous findings [10, 11]. The UTR regions are also thought to be crucial for forming the higher order RNA structure of BTV ssRNA segments. For other segmented ssRNA viruses, such as influenza virus [18–22] and the phi6 bacteriophage [23], the hierarchical intermolecular interactions between segment structures have been implicated in facilitating the efficient packaging of the viral genome.

Based on this, we performed subsequent *in vitro* studies targeting predominantly S10 ssRNA and other three smaller ssRNA segments (S7, S8 & S9) in order to facilitate the identification of supramolecular complexes and their disruption by antisense ORNs by EMSA. In particular, we examined co-transcription reaction products of ssRNAs S7-S10 in different combinations since it would allow *de novo* interactions between different transcripts as they were transcribed. Complexes with four segments were readily formed and were detectable in EMSA, indicating that such complexes possibly mimicked the phenomena of nascent BTV RNA interacting together through the RNA sorting and packaging signals prior to encapsidation. However, when various combinations of two or three segments were used, it was evident that both S7 and S10 RNAs not only interacted with each other but each also interacted with the other two small segments, S8 and S9. These data indicated that both S7 and S10 are important for formation of stable RNA complex and that the RNA complexes are formed through multi-segment interactions and not solely controlled by S10 as previously proposed. Further, two ORNs that targeted S10 3’ UTR (S10.2 and S10.5), could inhibit complex formation significantly between the S10 RNA and three other segments. Thus, these results indicated that when both ORNs bind in the S10 3’ UTR of S10, the predicted structures which consisted of hairpin loops, bulges and GC rich motifs were altered and affected RNA interactions. Further confirmation of importance of these ORNs regions were obtained by using two deletion mutants, ΔS10.2 and ΔS10.5 that lacked the corresponding ORN binding regions. Both mutants S10 exhibited significant reduction in RNA complex formation, which suggested that either the deleted sequences may form a part of the interaction site of other segments or the deletions might have perturbed the secondary structure of these regions, both of which are located in the hairpin loop and double-stranded region of hairpin stem. The importance of these structured motifs at the 3’ UTR was then demonstrated by using substitution mutations of five or six nucleotides to recover viable virus by reverse genetics, and the results showed mutations were highly lethal to virus viability. Changes in these sequences might have triggered conformational changes resulting in the loss of ssRNA recruiting function of S10 during capsid assembly. Interestingly, the interchange of 3’UTRs between S10 and S8 RNA segments found to be non-functional and had abrogated virus recovery. This may signify the need for segment specific sequences to trigger intramolecular interactions in individual segments itself and conformational changes on the RNA structure prior to interactions and base-pairing between segments which was abolished when the 3’UTR was removed. Most likely, 3’UTRs act as part of the secondary structure presented by the entire genomic segment, rather than as a linear sequence. This is consistent with data obtained on interchanging packaging signals in the 3’UTR of influenza A virus [24].

The data obtained from a series of *in vitro* and *in vivo* studies confirmed that small RNA nucleotides interfere in the recruitment and packaging of the ssRNA genomic segments and that genome packaging in this segmented dsRNA virus occurs via the formation of supramolecular complexes generated by the interaction of specific sequences located in the 3’ UTRs. Our data also indicate that RNA segment sorting occurs via specific interactions among the different segments followed by the supramolecular complex formation and packaging by the assembling core.

Reverse complementary or “antisense” oligonucleotides have been used extensively in recent years to study virus life cycles, including insight into RNA packaging signals, in addition their potential as antiviral molecules has also been demonstrated for a number of viral targets [13, 19, 25–31]. Our study, however, is the first to use ORNs as a tool for understanding dsRNA virus packaging and this has potential as a therapeutic strategy. Furthermore, the approaches used here to identify the possible location of an RNA packaging signal in the smallest segment of BTV can be applied to packaging signal analysis of related dsRNA viruses. These signals are a potential target for future research of BTV antivirals and could pave the way for the development of a small molecule based therapeutics to control this economically important virus.

## Materials and Methods

### Cells and virus

Bluetongue virus serotype 1 (BTV-1) South African reference strain was plaque purified and amplified in BSR cells, a BHK 21 clone derivative of baby hamster kidney cells (American Type Culture Collection) grown in Dulbecco modified Eagle medium containing 5% fetal calf serum (FCS) penicillin, streptomycin and amphotericin B at 35°C with 5% CO_2_. Virus stocks were maintained by infecting BSR cells at multiplicity of infection (MOI) of 0.1 and harvested at 48–72 hpi.

### Plasmids, mutagenesis and RNA synthesis

T7 transcripts were generated from exact cDNA copies of BTV-1 genome segments 7, 8, 9 and 10 (GenBank accession numbers FJ969719–FJ969728), flanked by T7 promoter and specific restriction enzyme sites [12]. For the generation of S10 RNA deletion mutants, two S10 deletion constructs corresponding to the target sequences of S10.2 (39 nts) and S10.5 (34 nts) ORNs were generated by polymerase chain reaction (PCR) through site-directed mutagenesis [32]. Amplicons were then treated with DpnI to digest the parental plasmid prior to transformation into competent cells. For the generation of four S10 RNA substitution mutants S10.2_**713–718**_ **,** S10.2_**725–730**_, S10_**728-732**_ and S10.5_**743–748**_ site directed mutagenesis was performed by overlapping PCR using S10 specific primers. Deletion and interchanging 3’UTRs of S8 and S10 were also generated by overlapping PCR followed by Dpn 1 treatment. Capped BTV RNA transcripts for *in vitro* translation assay were generated using mMESSAGE mMACHINE Kit (Ambion) as described previously [12]. For generation of uncapped ssRNA for cell-free assembly, linearized DNA were incubated at 37°C for 2 h with 40 U of T7 RNA polymerase (Thermo Scientific), 50 mM DTT, 0.5 mM each rNTP and 10 U RNase inhibitor (Thermo Scientific).

### Design of antisense oligoribonucleotides with 2′O-methyl modifications based on prediction of ssRNA structures

A series of thirteen antisense oligoribonucleotides (ORNs) were designed to hybridize either the 5’UTR including the AUG initiating codon, the internal coding region or the 3’ UTR of segments S1, S9 and S10 (Table 1). These ORNs were modified at the ribose with 2’O-methyl group (Integrated DNA Technologies) and named by their target position in each segment (Fig 1). A scrambled (SCR) sequence of 30 nt, was included as specificity control. The scrambled sequence was verified by NCBI-BLAST software (http://blast.ncbi.nlm.nih.gov/) to prevent any possible match in the BTV genome or the host cellular RNAs. For the design of the ORN target sites the software Mfold (http://rna.tbi.univie.ac.at/) and RNAfold (http://rna.tbi.univie.ac.at/cgi-bin/RNAfold.cgi) were used to predict the secondary structure and folding pattern of each RNA segments in the context of a full-length segment. OligoAnalyzer (http://eu.idtdna.com/calc/analyzer) was used to analyse each ORNs to avoid structures that might prevent its base-pairing to target RNA (perfect hairpin, self-dimerization and melting temperatures).

### Optimization of inhibitory conditions of 2′OMe ORNs and challenge with BTV-1

To determine the optimal inhibitory condition for each ORNs, a concentration range (0.5, 1.5 and 2.5 μM) of S10 AUG, S10 3’ UTR and SCR were transfected to BSR cells using Lipofectamine 2000 (Life Technologies). After 3 h incubation, the cells were infected with BTV-1 at MOI 0.1 for 1 h. The inoculum was removed by 3 washes with low pH medium (DMEM-HCl, pH 6) to inactivate free virus, twice with normal medium to restore pH and incubated with DMEM supplemented with 1% FCS and the appropriate ORNs for one virus replication cycle of 16–18 h. Cells were harvested and the virus titre was analysed by plaque assay. The virus yield was calculated as the mean of plaque forming units per ml (PFU/ml) of three independent transfection assays with each 2′OMe ORNs and expressed as the relative PFU/ml of BTV1 transfected without ORNs, consider as 100%. Cytotoxicity was determined by cell staining at the end of the treatment. The optimal concentration for the ORNs was 1.5 μM.

### *In vitro* translation in the presence of 2’OMethyl ORN

Different concentration range (0.5, 2 and 4 μM) of ORNs S1 AUG, S1.3’, S9 AUG, S9.1, S9.2, S10.1, S10.2, S10.3, S10.5, S10 AUG or Scr were incubated with BTV transcripts (300 ng) for 20 min at 37°C and added to a reaction mix containing 7.5 μl of nuclease-treated rabbit reticulocyte lysate (RRL, Promega), 1 mM amino acid mix minus methionine and 6 μCi ^35^S-methionine. Translation reactions were incubated at 30°C for 90 min and treated with 1 μl of 1μg/μl RNase A for 10 min at room temperature. Labelled proteins were quantified by densitometry using PhosphorImager screen. The inhibition of BTV protein expression was calculated relative to the control lacking ORNs. The experiment was repeated at least three times.

### *In vitro* transcription for RNA-RNA interaction assays, RNA-RNA interaction in the presence of ORN and electrophoretic mobility shift assay

For RNA-RNA interactions of individual RNA segments, 1 μg of linearized plasmid was transcribed in a buffer containing 40 mM Tris–HCl pH 7.5, 10 mM MgCl2, 20 mM NaCl2, 3 mM spermidine, 50 mM DTT, 5 mM each rNTPs, 10 U RNase inhibitor and 40 U of T7 RNA polymerase (Thermo Scientific) for 3 h at 37°C followed by RNase free DNase 1 treatment. Transcribed RNAs were extracted by standard phenol-chloroform method and re-suspended in RNase free water. RNAs were individually heated at 80°C for 1 min, ice chilled and mixed in pairs in folding buffer (50 mM Na cacodylate pH 7.5, 300 mM KCl and 10 mM MgCl_2_) [33] and RNA–RNA complexes were allowed to form for 90 min at 30°C and immediately analysed by electrophoresis in 1% agarose gel supplemented with 0.1mM MgCl_2_. Electrophoresis gel was run for 180 min at 150 V in TBM buffer (45 mM Tris, pH 8.3, 43 mM boric acid, 0.1 mM MgCl_2_) and stained with 0.01% (w/v) ethidium bromide. The integrity of transcribed RNA was checked by denaturing gel electrophoresis.

For co-transcription experiments, 150 ng linearized plasmid of each segments (S7-S10) were transcribed either in pairs or combinations of 3 to 4 plasmids (S7, S8, S9 and S10 or S10 mutants). RNA transcription was carried out in the same condition as individual RNA segments. Immediately after transcription and DNase 1 treatment, the reaction was analysed on a 1% agarose gel as described above. The percentage of the retarded RNA in each lane was determined against the total mass of input RNA (%) by densitometry (Gene Tools, Syngene). For RNA complex inhibition assay with ORNs, the simultaneous transcription of S7-S10 (combination of 3 or 4) was performed in the presence or absence of 20 pmol of S10.1, S10.2, S10.4, S10.5 and Scr ORNs and analysed as described above. Non-specific yeast tRNA (20 and 50 pmol) was incorporated in the co-transcription reaction as a control. Quantification of intermolecular RNA complex was performed as described above.

For RNA-ORN hybridization assay, 10pmol of S9 AUG, S9.2, S10 AUG, S10.2, S10.3, S10.5 and Scr ORNs were 3’ end labelled with 10 μCi [^32^P]pCp (Perkin Elmer) with T4 RNA ligase (Thermo Scientific) in T4 RNA ligase buffer and incubated at 4°C overnight. Unincorporated ^32^P was removed by exclusion chromatography (Illustra Microspin G-25 column, GE Healthcare). Prior to hybridization, unlabelled S10 RNA was denatured at 80°C for 1 min, immediately chilled and then mixed with folding buffer (50 mM sodium cacodylate pH 7.5, 100 mM KCl and 10 mM MgCl_2_). RNA-ORN hybridization was performed with 0.5pmol of pre-folded S10 RNA annealed with ^32^P labelled ORNs (1, 2 and 5 pmol of S9 AUG, S9.2, S10 AUG, S10.2, S10.3, S10.5 and Scr ORNs) in folding buffer in 10 μl final volume [34]. The complex was allowed to form for 30 min at 30°C followed by electrophoresis in 4% native acrylamide gel at 4°C for 50 min at 150V in TBM buffer, dried and exposed by autoradiography.

### Cell-free *in vitro* packaging assay

The cell-free system for BTV was carried out as described [6] with some modifications. Briefly, VP1, VP4 and VP6 were synthesized from RRL system followed by incubation with the complete set of 10 full-length (300ng each) uncapped ssRNAs with or without 20 pmol S10.1, S10.2, S10.4, S10.5, S10 AUG and Scr ORNs. *In vitro* synthesized VP3 and VP7 were then added to the mixture and further incubated to allow viral core assembly. After eliminating unpackaged RNA by RNase One (Promega) digestion, the assembled particles in the reaction mixture were isolated by a 15% to 65% continuous sucrose gradient followed by fractionation as described previously [6]. For positive control, S10.2 and S10.5 ORN gradients, packaged RNAs were extracted from fractions 5, 6 and 7 and analysed by denaturing 1% agarose gel electrophoresis to identify the packaged 10 ssRNAs [6]. Only fraction 6 was collected for samples with S10.1, S10.4, S10.5, S10 AUG and Scr (packaged ssRNAs are previously shown to be present at this fraction) [6]. For analysis of *in vitro* incorporated proteins, the *in vitro* synthesized viral proteins were radio labelled with ^35^S-methionine, analysed in 9% SDS-PAGE and detected by autoradiography.

### Reverse genetics

To generate the virus with S10 mutants (S10.2_**713–718**_ **,** S10.2_**725–730**_, S10_**728-732**_ and S10.5_**743–748**_, and chimeric S10 and S8) BSR cells were transfected with mutated S10 ssRNA together with the remaining 9 BTV-1 ssRNAs as described previously [12, 35]. For combined chimeric S10 and S8, BSR cells were transfected with mutated S10 ssRNA together with the remaining 8 BTV-1 ssRNAs. Replication of recovered viruses was visualised by crystal violet staining. Virus recovery was quantified by qRT-PCR using specific BTV genomic primers as previously described [10]. To confirm the recovery of mutant virus, genomic dsRNAs were purified from the infected cells, reverse transcribed and the mutated sequences of S10 was confirmed by nucleotide sequencing (Source Bioscience).

## Supporting Information

S1 Fig Secondary structure of S10 3’ UTR from RNAfold. The binding region of S10 ORNs (S10.1, S10.2 and S10.5 ORNs) in 3’UTR are coloured.
(TIF)

S2 Fig Secondary structure of S9 3’ UTR from RNAfold. The binding region of S9 ORNs (S9.1 and S9.2) in 3’UTR are highlighted bold.
(TIF)

S3 Fig Secondary structure of S1 3’ UTR from RNAfold. The binding region of S1 ORN (S1.3’) in 3’UTR is highlighted bold.
(TIF)

S4 Fig ORN cell toxicity assay on BSR cells. **A**. Representative examples of BSR cells transfected with different concentration of Scr ORN and stained with crystal violet after 48h showing no sign of cell toxicity. **B**. For comparison, BSR cells transfected with 2.5uM of Scr were infected with 0.1 MOI of BTV 1 showing non-inhibition of virus replication.
(TIF)

S5 Fig RNAfold secondary structure of 3’ UTR of S10 with deletion mutations. The binding region of the S10.2 and S10.5 ORNs are coloured. Predicted secondary structure of S10 with deletion corresponding to S10.2 and S10.5 binding regions (ΔS10.2 and ΔS10.5) are shown.
(TIF)

S6 Fig RNAfold secondary structure of 3’ UTR of S10 with substitution mutations. Substituted regions of the three mutants are coloured. 3’ UTR secondary structure of these mutations predicted with RNAfold are also shown.
(TIF)

S7 Fig Effect of ORN on RNA packaging in *cell-free* assembly assay with S10.1 and S10 AUG ORN. ^35^S-labelled *in vitro* assembled BTV complexes were fractionated in a continuous sucrose gradient. Fraction #6 from *cell-free* assembly (CFA) reactions in the absence (+control) or presence of 20 pmol S10.1 and S10.AUG ORNs as indicated were analyzed on 1% denaturing agarose gel.
(TIF)

S8 Fig Synthesized ^35^S labelled BTV core and transcriptase proteins. Fractions 6 and 10 of the complete BTV subcore and transcription complex ^35^S-labelled protein profile of *in vitro* translation of assembled ssRNA in the presence or absence of ORN were analyzed on 8% SDS-PAGE gel. Molecular size of each BTV protein and marker are indicated.
(TIF)

S9 Fig Secondary structure of S8 3’ UTR. Secondary structure of S8 3’UTR from RNAfold.
(TIF)

S10 Fig The binding affinity of S10 and ORNs. P^32^ labelled S9 AUG, S9.2, S10 AUG, S10.2, S10.3, S10.5 and Scr ORNs (1, 2 and 5 pmol) were hybridized to 0.5pmol of S9 and S10 RNA in a folding buffer and incubated for 30 min at 30°C. The complex was analysed on 4% native acrylamide gel followed by autoradiography.
(TIF)

S11 Fig Representative examples of plaque assay from reverse genetics done with mutant S10. BSR monolayer cells were transfected with mutant S10 together with 9 wild-type ssRNAs (S1-S9) for 3h and overlayed with 1% agarose with DMEM and 1% FCS. At 72hpt the monolayer was fixed with 10% formaldehyde and stained with crystal violet as described in Materials and Methods.
(TIF)
