# Supplementary figures and images for: Disruption of Specific RNA-RNA Interactions in a Double-Stranded RNA Virus Inhibits Genome Packaging and Virus Infectivity
Source: PLoS Pathog. 2015 Dec 8;11(12):e1005321. doi: 10.1371/journal.ppat.1005321 (PMC4672896; doi:10.1371/journal.ppat.1005321)

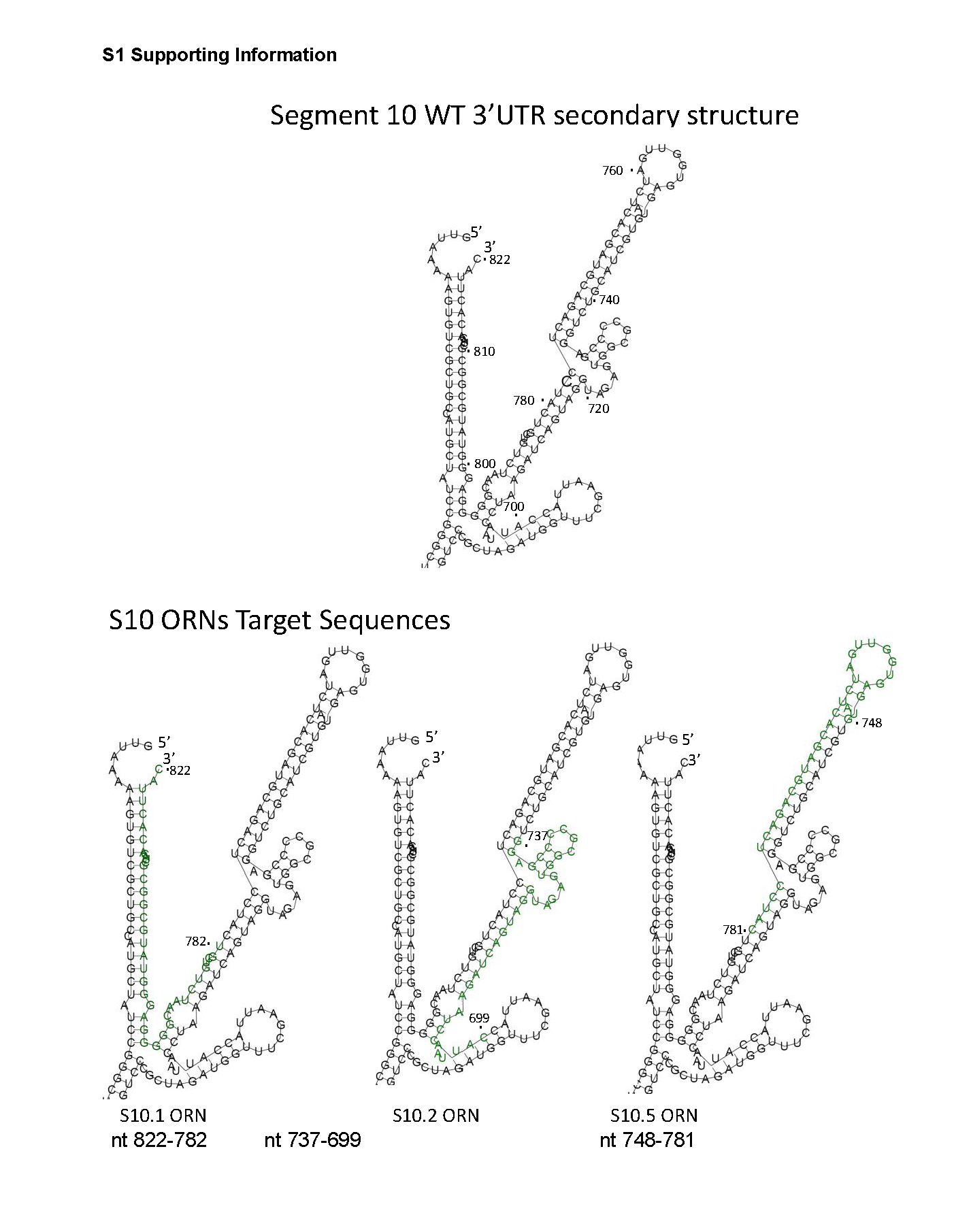

Supplement: S1 Fig — The binding region of S10 ORNs (S10.1, S10.2 and S10.5 ORNs) in 3’UTR are coloured. (TIF) [file ppat.1005321.s001.tif]

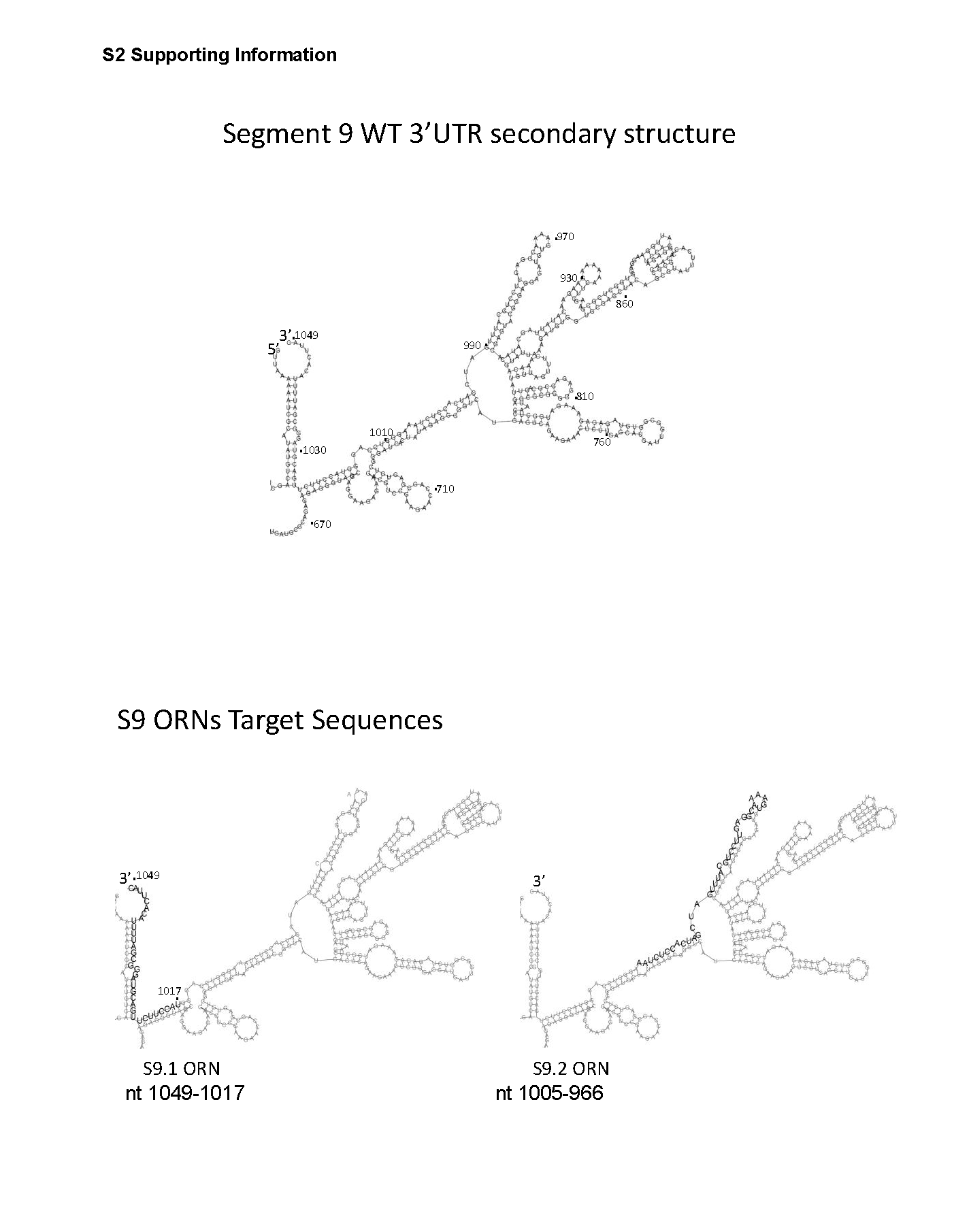

Supplement: S2 Fig — The binding region of S9 ORNs (S9.1 and S9.2) in 3’UTR are highlighted bold. (TIF) [file ppat.1005321.s002.tif]

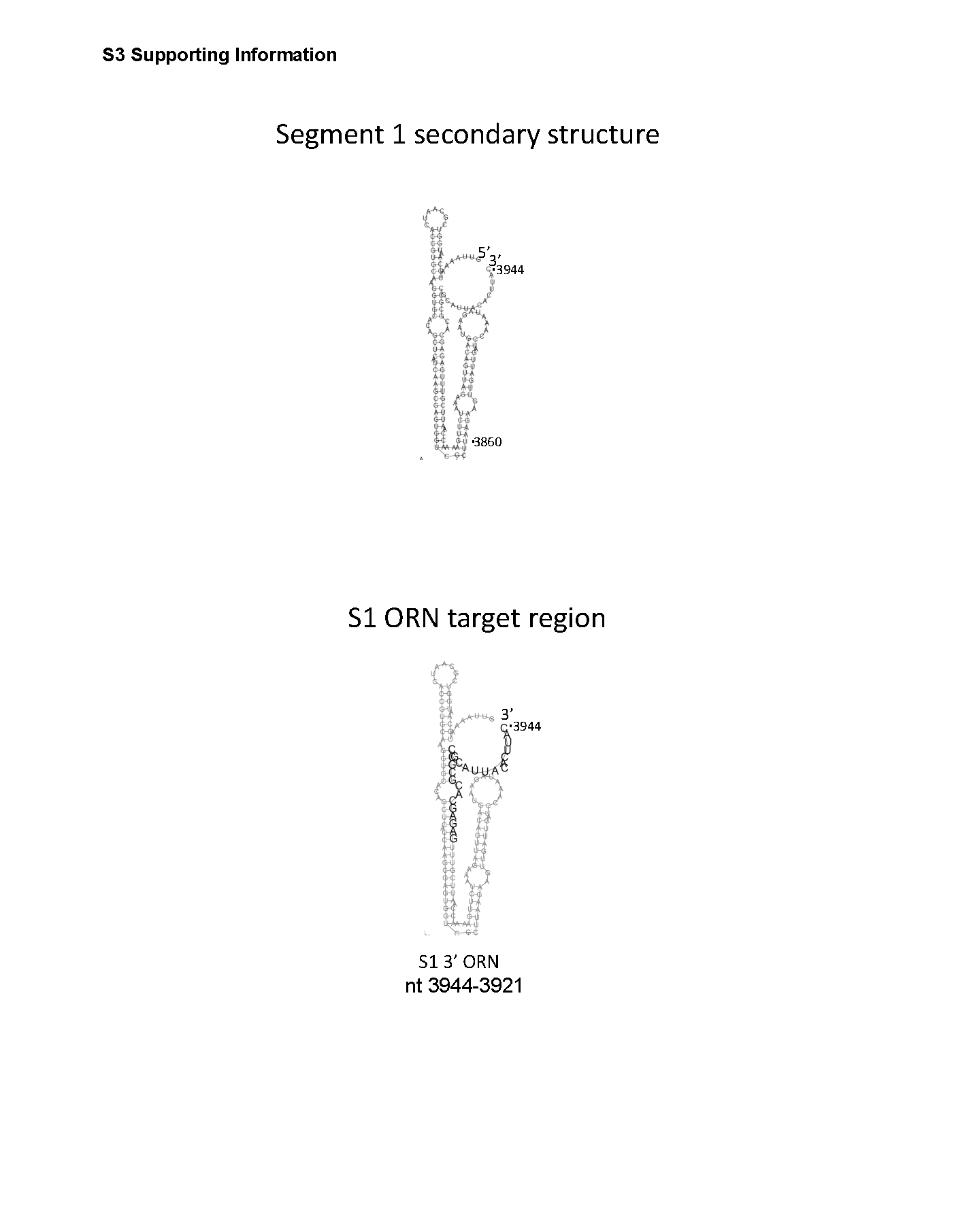

Supplement: S3 Fig — The binding region of S1 ORN (S1.3’) in 3’UTR is highlighted bold. (TIF) [file ppat.1005321.s003.tif]

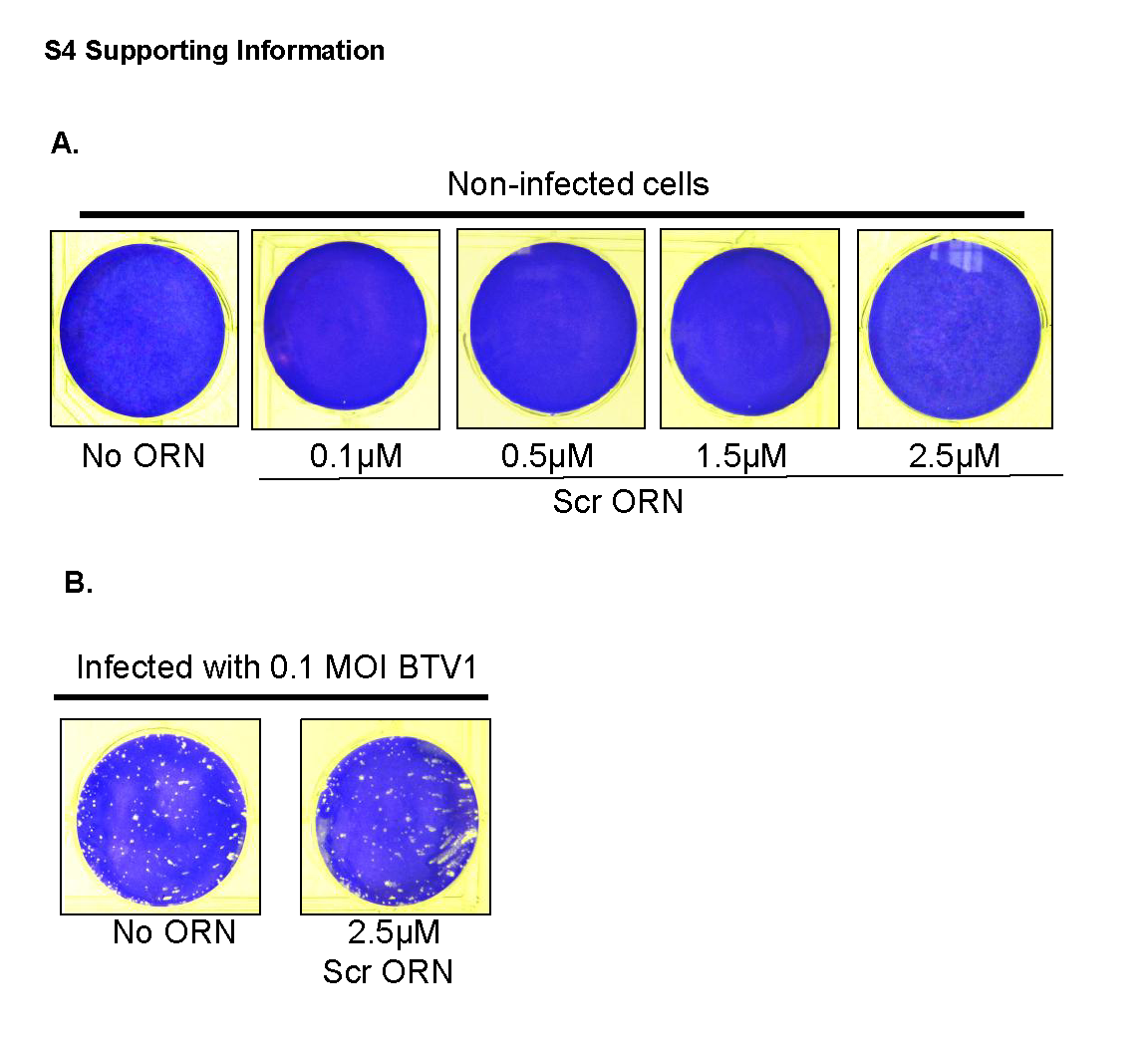

Supplement: S4 Fig — A. Representative examples of BSR cells transfected with different concentration of Scr ORN and stained with crystal violet after 48h showing no sign of cell toxicity. B. For comparison, BSR cells transfected with 2.5uM of Scr were infected with 0.1 MOI of BTV 1 showing non-inhibition of virus replication. (TIF) [file ppat.1005321.s004.tif]

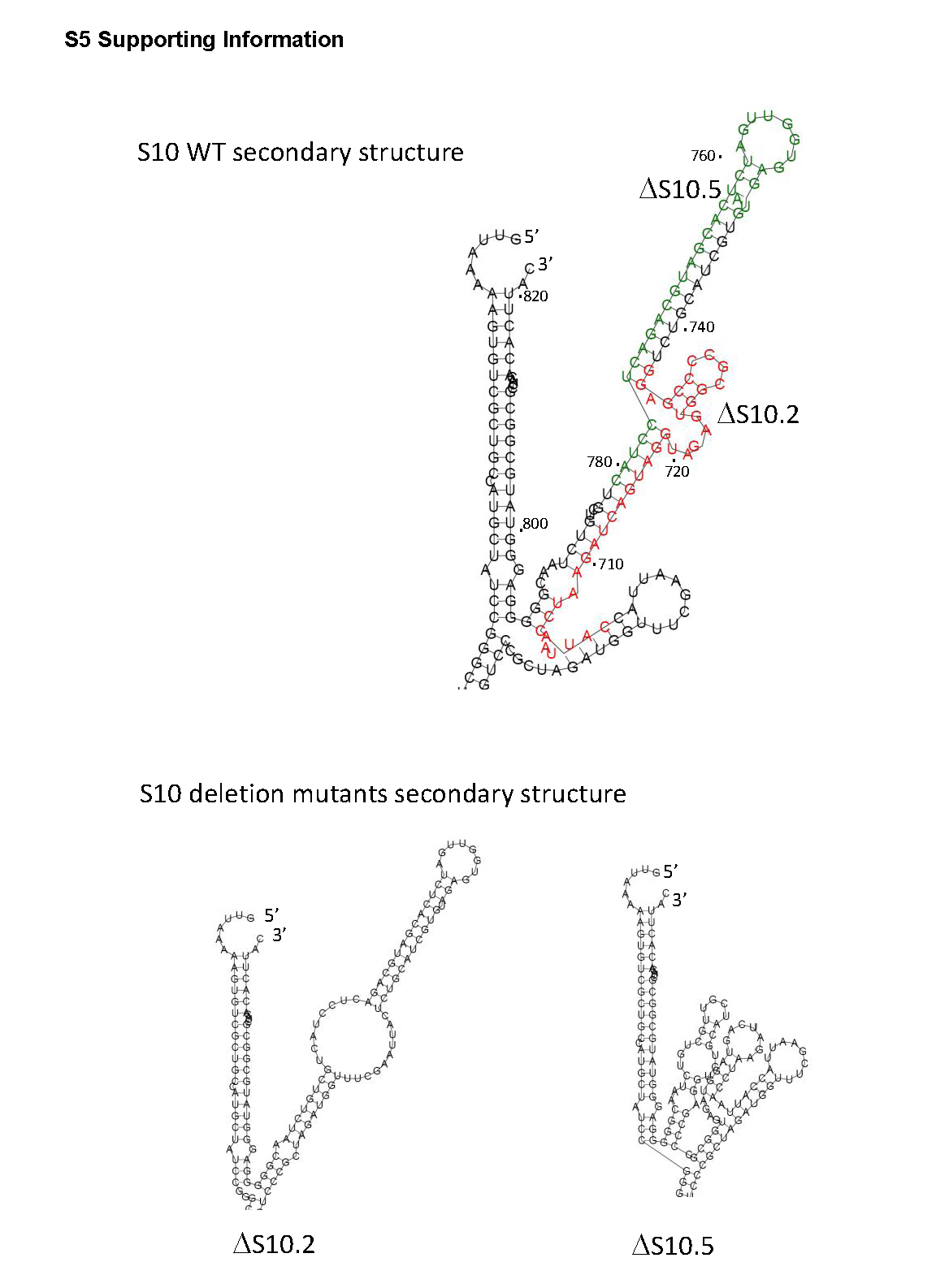

Supplement: S5 Fig — The binding region of the S10.2 and S10.5 ORNs are coloured. Predicted secondary structure of S10 with deletion corresponding to S10.2 and S10.5 binding regions (ΔS10.2 and ΔS10.5) are shown. (TIF) [file ppat.1005321.s005.tif]

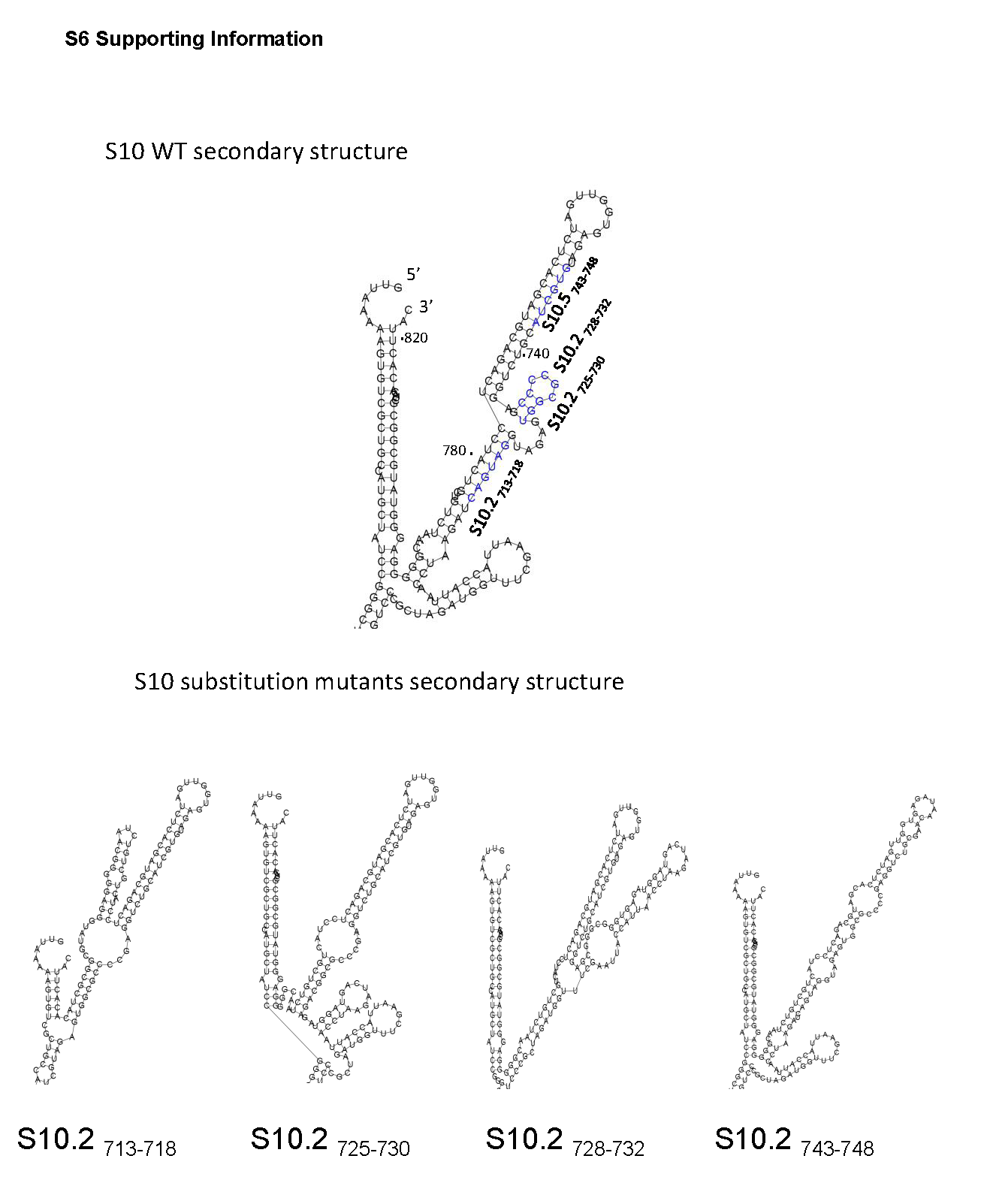

Supplement: S6 Fig — Substituted regions of the three mutants are coloured. 3’ UTR secondary structure of these mutations predicted with RNAfold are also shown. (TIF) [file ppat.1005321.s006.tif]

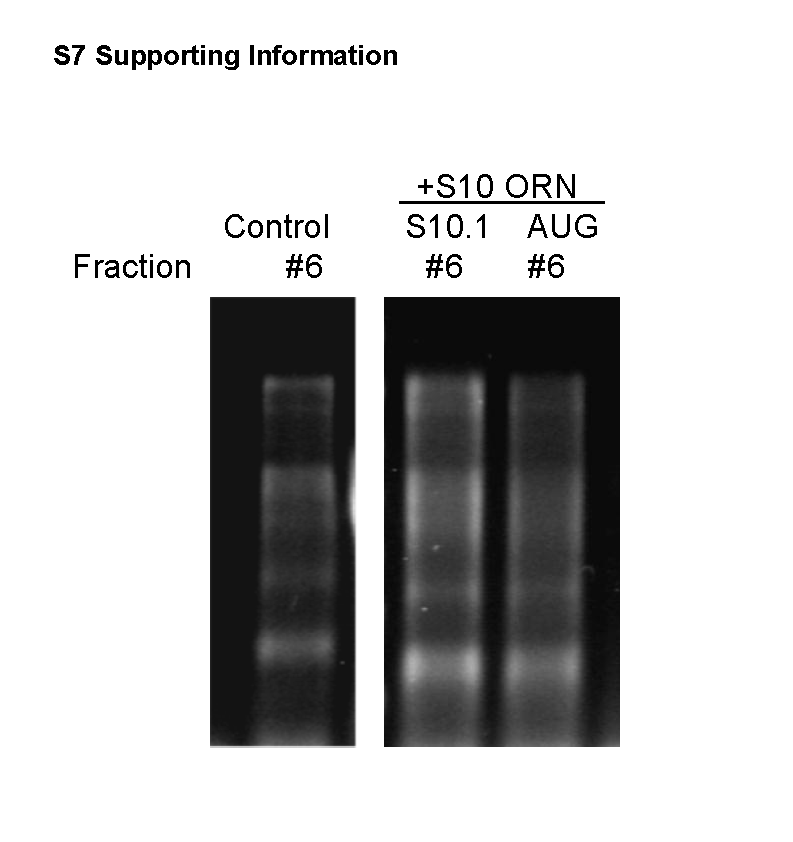

Supplement: S7 Fig — 35S-labelled in vitro assembled BTV complexes were fractionated in a continuous sucrose gradient. Fraction #6 from cell-free assembly (CFA) reactions in the absence (+control) or presence of 20 pmol S10.1 and S10.AUG ORNs as indicated were analyzed on 1% denaturing agarose gel. (TIF) [file ppat.1005321.s007.tif]

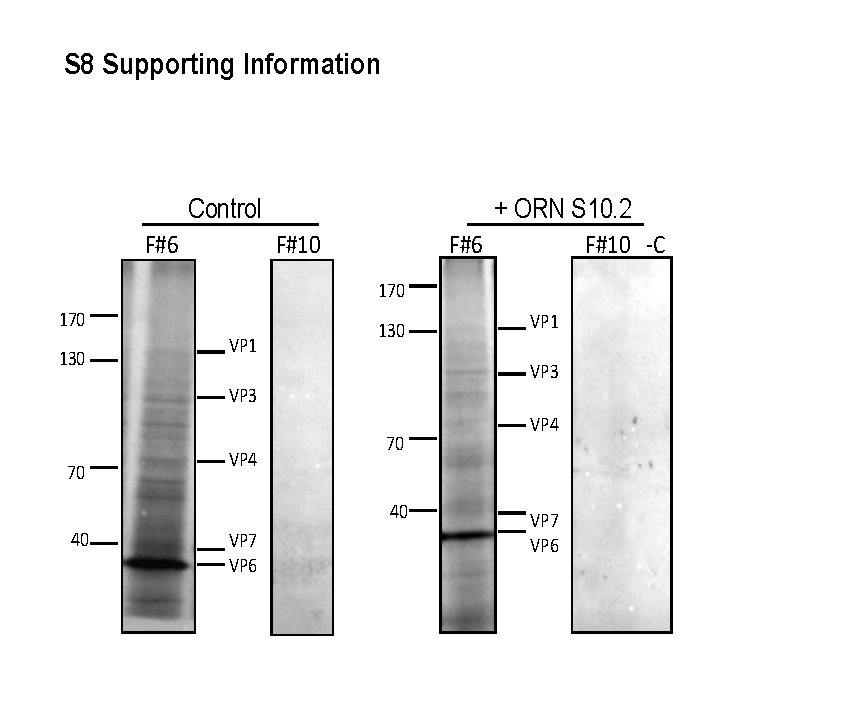

Supplement: S8 Fig — Fractions 6 and 10 of the complete BTV subcore and transcription complex 35S-labelled protein profile of in vitro translation of assembled ssRNA in the presence or absence of ORN were analyzed on 8% SDS-PAGE gel. Molecular size of each BTV protein and marker are indicated. (TIF) [file ppat.1005321.s008.tif]

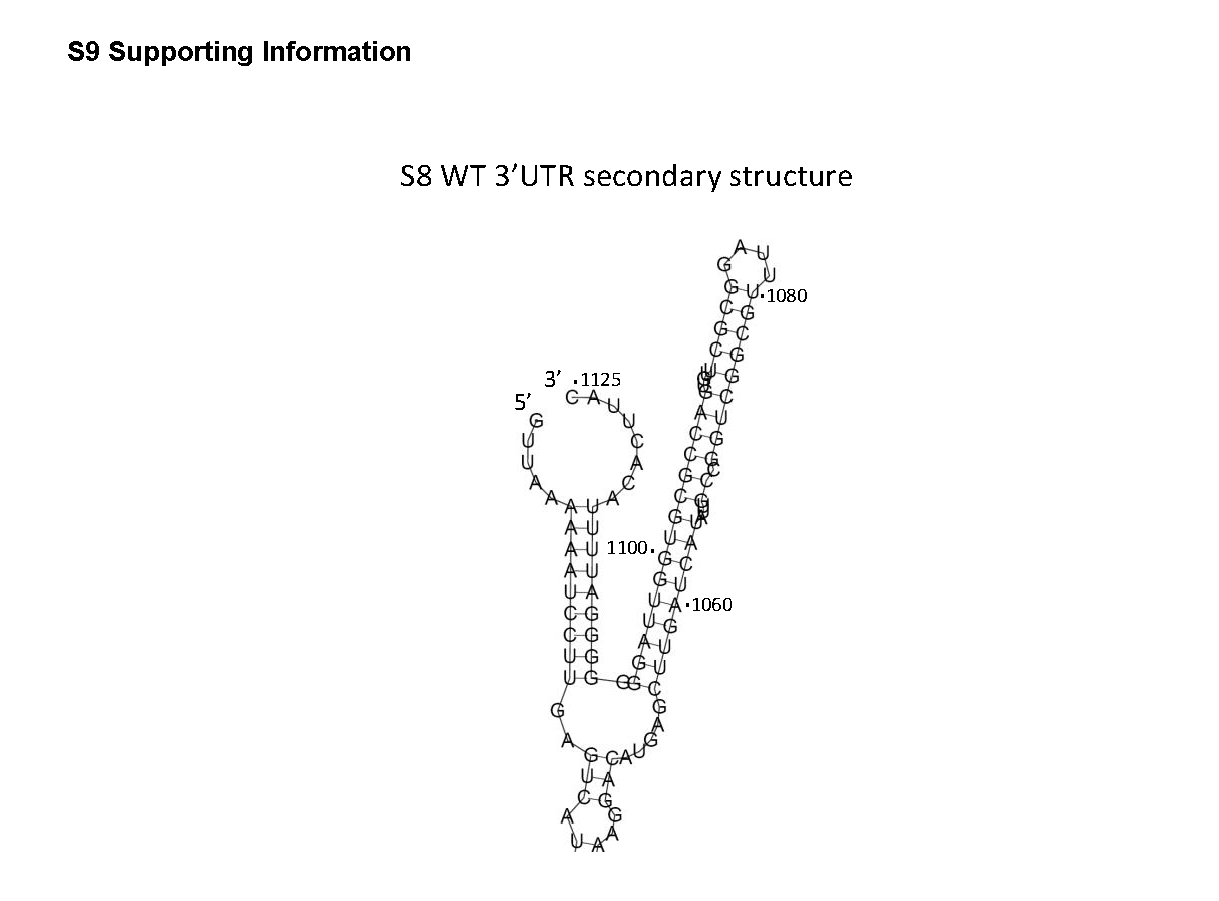

Supplement: S9 Fig — Secondary structure of S8 3’UTR from RNAfold. (TIF) [file ppat.1005321.s009.tif]

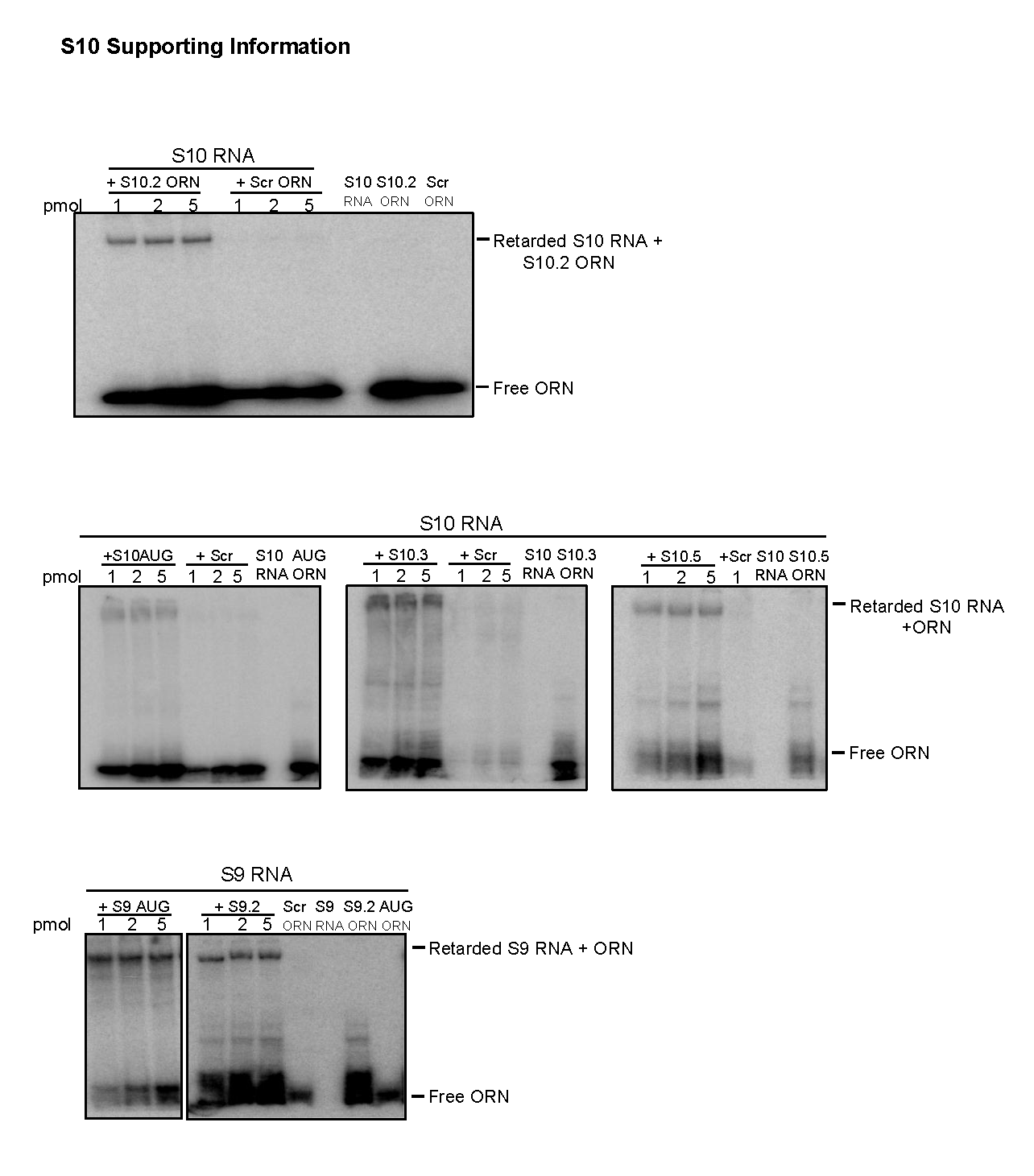

Supplement: S10 Fig — P32 labelled S9 AUG, S9.2, S10 AUG, S10.2, S10.3, S10.5 and Scr ORNs (1, 2 and 5 pmol) were hybridized to 0.5pmol of S9 and S10 RNA in a folding buffer and incubated for 30 min at 30°C. The complex was analysed on 4% native acrylamide gel followed by autoradiography. (TIF) [file ppat.1005321.s010.tif]

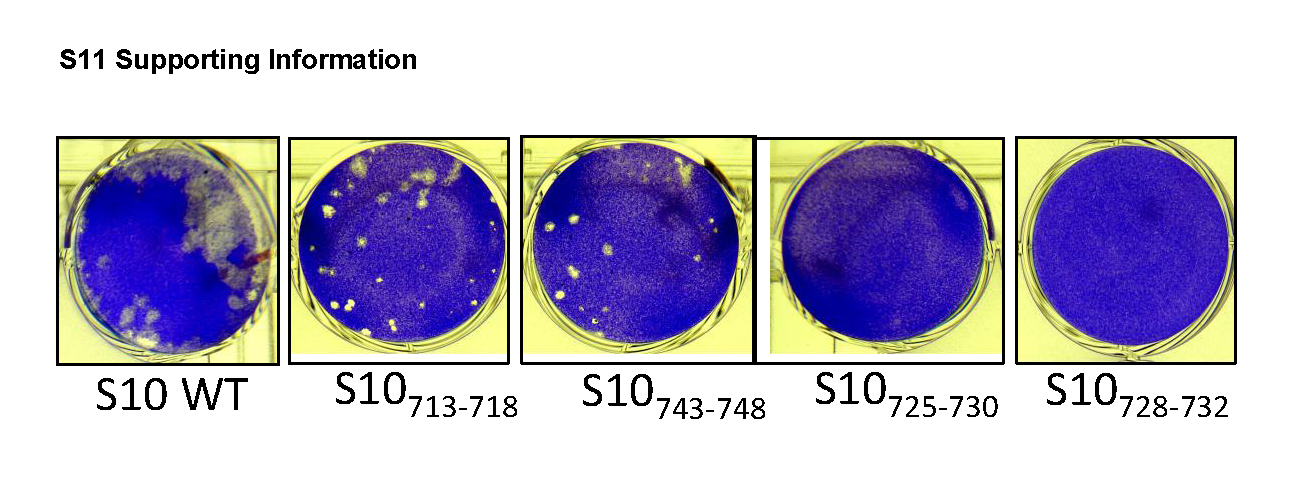

Supplement: S11 Fig — BSR monolayer cells were transfected with mutant S10 together with 9 wild-type ssRNAs (S1-S9) for 3h and overlayed with 1% agarose with DMEM and 1% FCS. At 72hpt the monolayer was fixed with 10% formaldehyde and stained with crystal violet as described in Materials and Methods. (TIF) [file ppat.1005321.s011.tif]
